# Robot tool use: A survey

**DOI:** 10.3389/frobt.2022.1009488

**Published:** 2023-01-16

**Authors:** Meiying Qin, Jake Brawer, Brian Scassellati

**Affiliations:** Yale Social Robotics Lab, Department of Computer Science, Yale University, New Haven, CT, United States

**Keywords:** survey, robot tool use, human tools, manipulation, affordance

## Abstract

Using human tools can significantly benefit robots in many application domains. Such ability would allow robots to solve problems that they were unable to without tools. However, robot tool use is a challenging task. Tool use was initially considered to be the ability that distinguishes human beings from other animals. We identify three skills required for robot tool use: perception, manipulation, and high-level cognition skills. While both general manipulation tasks and tool use tasks require the same level of perception accuracy, there are unique manipulation and cognition challenges in robot tool use. In this survey, we first define robot tool use. The definition highlighted the skills required for robot tool use. The skills coincide with an affordance model which defined a three-way relation between actions, objects, and effects. We also compile a taxonomy of robot tool use with insights from animal tool use literature. Our definition and taxonomy lay a theoretical foundation for future robot tool use studies and also serve as practical guidelines for robot tool use applications. We first categorize tool use based on the context of the task. The contexts are highly similar for the same task (e.g., cutting) in *non-causal tool use*, while the contexts for *causal tool use* are diverse. We further categorize causal tool use based on the task complexity suggested in animal tool use studies into *single-manipulation tool use* and *multiple-manipulation tool use*. Single-manipulation tool use are sub-categorized based on tool features and prior experiences of tool use. This type of tool may be considered as building blocks of causal tool use. Multiple-manipulation tool use combines these building blocks in different ways. The different combinations categorize multiple-manipulation tool use. Moreover, we identify different skills required in each sub-type in the taxonomy. We then review previous studies on robot tool use based on the taxonomy and describe how the relations are learned in these studies. We conclude with a discussion of the current applications of robot tool use and open questions to address future robot tool use.

## 1 Introduction

Many robots are designed to interact with objects in the environment. Recent advances grant robots the ability to perform various tasks ranging from everyday tasks, such as swiping a card (Sukhoy et al., 2012), to professional tasks that require high precision, such as robot surgery (Sarikaya et al., 2017).

Among these tasks, robot tool use is gaining increasing attention. Being able to use human tools such as screwdrivers and scissors can greatly expand the applicability of a robot. Household robots will be able to assist humans better by performing a wider range of tasks with everyday tools; robots in chemistry labs will be able to run more experiments by leveraging the lab tools; manufacturing robots will be able to complete more tasks by utilizing construction tools without the need for specialized grippers. In this survey, a *tool* refers to the object attached to a robot. A *manipulandum* refers to the object being manipulated by the tool. An *object* is an umbrella term to include both tools and manipulanda.

Robot tool use requires three skills. The first skill is perception. A robot should identify and localize tools and manipulanda from the environment. For example, to drive a slotted screw, the robot needs to align the slotted screwdriver with the screw. Inaccurate pose perception of the screw will lead to misalignment, resulting in the failure of the tool use action. To successfully drive a screw, position knowledge alone is insufficient. In the above example, the tip of the screwdriver should both be at the position of the top of the screw, and oriented in a way that the flat tip of the screwdriver is aligned with the slot of the screw. Though challenging, the perception requirement is not unique to tool use. General robot manipulation also requires similar perceptual capabilities (Li et al., 2022).

**FIGURE 1:**
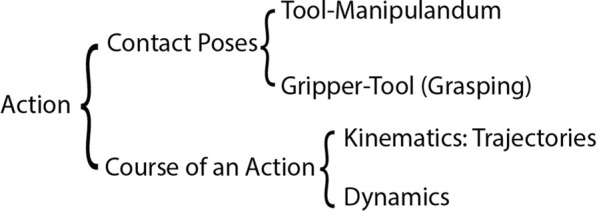
Components of an action. The diagram is adapted from Qin et al. (2021), CC BY.

The second skill of robot tool use is manipulation. Manipulation skills focus on how to realize the required kinematics and dynamics of tool use actions. The actions include two components as defined in Qin et al. (2021) and demonstrated in Figure 1: the contact poses and the course of the action. The contact poses include tool-manipulandum contact poses and gripper-tool contact poses (i.e., grasping). These poses consider both the translational (e.g., the tip of the pen should contact a point on the paper) and the rotational (e.g., the pen should contact the paper close to perpendicular to the plane of the paper, rather than parallel to it) relations of the tool and the manipulanda, or the tools and the gripper. Manipulation also encompasses both the tool trajectory (i.e., a time series of poses of a tool) and dynamics (i.e., the forces required for successful tool use). Though the manipulation skills required in tool use tasks may share similarity with general manipulation tasks, tool use additionally requires that a robot should update its body schema when a tool is held (Stoytchev, 2003). For example, general manipulation tasks consider exerting certain forces at the end-effector, while tool use tasks concern how the forces be generated at the tool rather than at the end-effector.

The third skill of robot tool use is high-level cognition. This includes reasoning and planning tool use actions given the tasks and available tools. For example, a robot may need to reason about how to grasp the tool to facilitate tool use, and determine the tool-manipulanda contact pose, the trajectory, and the force needed to use tools successfully. The robot also reasons about using a novel tool when learned tools are unavailable. Moreover, the robot should plan to use multiple tools to achieve a goal. Neurological evidence also supports that different cognitive processes were involved when a human uses a tool compared with separate hand and tool actions (Cabrera-Álvarez and Clayton, 2020).

The unique skills required by tool use distinguish tool use from general manipulation tasks (for a review on robot manipulation, see Mason, 2018; Kroemer et al., 2021). In the following sections, we start with the discussion of what is tool use in robotics. Defining robot tool use is not a trivial task and we gain insights in animal studies which has a long history of studying tool use, especially how they distinguish tool use from general manipulations in animal behaviors. In Section 2, we present key results in animal studies and describe our definition of robot tool use. The definition not only sets a boundary for tool use, but also illuminates important skills of robot tool use. Moreover, researchers in animal studies also categorized different types of tool use and summarized the different skills required in each type of tool use. With this knowledge, we compile a tool use taxonomy in Section 3 and describe the skills required by different sub-types of tool use tasks in Section 4. The definition and taxonomy set a theoretical foundation for robot tool use, which is currently lacking in robotics research and acts. They also serve as a practical guideline for future applications. In Section 5, we organize robot tool use studies based on our taxonomy and focus on 1) the unique challenges of tool use tasks compared with general manipulation tasks in learning the required skills and 2) current advancements in how the skills are learned. We conclude this survey by discussing current applications of robot tool use and identifying the open challenges remaining in robot tool use.

## 2 Definition of robot tool use

Defining robot tool use is necessary to understand the uniqueness of tool use as described in Section 1. Although the concept of robot tool use is intuitive, it is challenging to provide a precise definition to show clear distinctions from general manipulations. For example, one may consider robot tool use as robots using objects in the environment to achieve a goal. Under this definition, robots using refrigerators to store food should count as robot tool use. Along this line of logic, robots using the floor to support themselves should also be considered as robot tool use, which is counter-intuitive.

Researchers in animal tool use also encountered similar challenges. Tool use was initially considered a unique behavior only shown in humans (Oakley, 1944). With observations of tool use in animals, researchers debated which instances are genuinely tool use. For example, some may argue that a chimpanzee using a rock to crack a nut is tool use but cracking a nut against an anvil is not, while others may consider both cases are tool use. The debate urged a precise definition of animal tool use. In this section, we present key results in animal studies, gain insights from their argument, describe our definition of robot tool use, and explore the necessary aspects of tool use based on our definition. We focus on the implications of these animal studies for robot tool use, rather than on the implications for animal cognition. Therefore, the review of animal tool use studies is not meant to be comprehensive.

Van Lawick-Goodall (1970, p. 195) defined tool use as “the use of an external object as a functional extension of mouth or beak, hand or claw, in the attainment of an immediate goal,” emphasizing the goal-oriented and functional character of tool-use. Alcock (1972, p. 464) revised the definition by specifying the kind of objects that can be used as tools and identifying the scope of the goals: “Tool-using involves the manipulation of an inanimate object, not internally manufactured, with the effect of improving the animal’s efficiency in altering the form or position of some separate object.”

Beck (1980) identified a number of shortcomings with these definitions. First, only objects that are portable and manipulable should be considered as tools. Under this definition, dropping a stone on an egg would be considered an example of tool use, but pounding a fruit on a tree would not be. The latter case is considered proto-tool-use (Parker and Gibson, 1977). Second, an agent should understand the connection between the goal and the tool. Otherwise, the conditioned behavior of a rat pressing a lever in a Skinner box would be considered tool use, and Beck considered this inappropriate. Third, the tool need not be externally manufactured to the agent using it nor inanimate. Researchers observed that captive apes threw feces toward human intruders, and a chimpanzee utilized the dead body of a colobus monkey to hit a conspecific, suggesting that a live ape could be utilized in a similar way. Beck argued that these behaviors should be considered tool use. Fourth, the goal of tool use can be extended beyond feeding or drinking to other goals such as self-maintenance. As a result, Beck (1980, p. 10) re-defined tool use as “the external employment of an unattached environmental object to alter more efficiently the form, position, or condition of another object, another organism, or the user itself, when the user holds or carries the tool during or just prior to use and is responsible for the proper and effective orientation of the tool.”

For decades, Beck’s definition has been accepted widely in the field of animal cognition and was even adopted in early robot tool use studies (e.g., Stoytchev, 2007). Two observations motivated St. Amant and Horton (2008) to propose a new definition of tool use. Krützen et al. (2005) reported that dolphins hold marine sponges in their rostrum in order to prevent potential injuries when probing for food. Breuer et al. (2005) observed that a wild gorilla tested the depth of water with a stick while it walked across a pond. These two behaviors fall outside of Beck’s definition of tool use since they do not involve altering the state of another object. St. Amant and Horton were also concerned about Beck’s definition that it over-emphasized peripheral aspects of tool use, such as the unattached property; an animal can use a stick that is still attached to a tree as a tool. Moreover, they argued that Beck’s definition is vague to determine whether a goal was achieved accidentally. They observed that purposeful behaviors require a continuum of control. Therefore, St. Amant and Horton (2008, p. 1203) re-defined tool use as “the exertion of control over a freely manipulable external object (the tool) with the goal of 1) altering the physical properties of another object, substance, surface or medium (the target, which may be the tool user or another organism) *via* a dynamic mechanical interaction, or 2) mediating the flow of information between the tool user and the environment or other organisms in the environment.” They elaborated that the interactions between tools and manipulanda should be dynamic. Under this definition, stacking boxes to reach bananas is not tool use since the interactions between boxes remains fixed once they have been stacked, while cracking a nut with rock is tool use because the interactions between the nut and the rock is constantly changing.

Shumaker and Walkup joined Beck to revise the Beck’s widely accepted definition and incorporated St. Amant and Horton’s argument: “the external employment of an unattached or manipulable attached environmental object to alter more efficiently the form, position, or condition of another object, another organism, or the user itself, when the user holds and directly manipulates the tool during or prior to use and is responsible for the proper and effective orientation of the tool.” (Shumaker et al., 2011, p. 36) We based our definition of robot tool use on this revised definition.

Other definitions of tool use in animal studies exist. Some may simply be shorter versions of these definitions (e.g., Chevalier-Skolnikoff, 1989; Matsuzawa, 1999). Others may disagree with the scope of tool use. For example, Asano (1994) did not restrict the tools to be something being held. This might result in the scope of tool use being overly broad since any behavior may eventually count as tool use, such as walking, which utilizes the ground as the “tool”. Lestel and Grundmann (1999) expands the scope of tool use even more by including abstract concepts such as culture as potential tools. These discussions may be too philosophical and lack operational details for robotics research.

We identify three essential points in these definitions. First, tool use must have a goal, despite a lack of consensus regarding a goal’s scope. Second, instead of achieving a goal through random exploration, an agent utilizing a tool should understand the connection between the goal and the behavior. Third, the tool should satisfy specific physical criteria, such as being freely manipulable. Based on these points, we define *robot tool use* as:

A robot attaches or secures to its end-effector an external, unanimated, freely available object or an object attached to another object, in order to achieve a goal of altering the state of another object, updating its own state, or other goals, through purposeful manipulations.

Our definition adopts Shumaker et al.‘s definition with minor modifications. First, we restrict the tools to be externally manufactured and unanimated. Unlike living creatures, a robot typically does not produce materials (e.g., feces, spider webs) from its body. We require the tools to be unanimated because an animated object that a robot would most likely manipulate is another robot. We consider this a better fit to the area of multi-agent systems rather than tool use since it involves synchronization and communication between robots. Second, we relax the interactions between the tool and object to be manipulated to be dynamic or static. Therefore, using a container to relocate other objects would count as tool use. Third, we relax the goal of tool use. The scope of the goals in animal tool use was summarized based on animal behavioral observations. Given that tool use in animals is structurally simple even in non-human primates (Fragaszy and Eshchar, 2017), the goals in the above definitions are restricted to altering the state of another object and updating one’s knowledge about the environment. In contrast, as robots often utilize human tools, robot tool use is motivated by the same goals for which these tools were designed, goals that can far exceed in scope and complexity those observed in animal studies. On the contrary, robots are required to utilize human tools. The design of human tools is more complex than those used by animals so that human tools may serve purposes beyond the goals identified in animal studies. Therefore, we prefer not to restrict the scope of the goal of robot tool use.

## 3 A taxonomy of robot tool use

Researchers of animal tool use recognizes that there are different types of tool use. For example, antlions mechanically throwing sand to capture preys in the same manner across all contexts is notably different from chimpanzees carefully adjusting sticks to fish termites even in the same context. Similarly, robot tool use also has many different types. A robot being pre-programmed to cut pizza with a particular knife is very different from it learning to adjust the gestures when presented with different knifes. In this section, we overview taxonomies proposed in animal and robotics studies, and present a taxonomy on robot tool use.

### 3.1 Taxonomy of animal tool use

Alcock (1972) proposed a dichotomy of tool use in animals: *stereotyped tool use* is seen mostly in invertebrates and fish and *flexible tool use* is typically seen in birds and mammals. Hunt et al. (2013) considered this dichotomy an accurate description of two fundamental types of tool use with different underlying processes, despite being oversimplified. Stereotyped tool use is inherited and animals only utilize tools in default ways in particular contexts. Examples of stereotyped tool use include antlions throwing sand to capture preys (Alcock, 1972). The species of antlions developed this behavior from the pre-existing non-tool use behavior of random sand flicking in order to maintain their pits. The tool use behavior of antlions throwing sand evolves as a phenotypic change in this species. As a result, these behaviors are widespread across the species of antlions and rarely vary within and across individuals.

In contrast, flexible tool use, which is also referred to as creative tool use, is learning-based that animals explicitly reasoning about the usages based on the context. It is this type of tool use that some believe signals intelligence (Call, 2013) and interests researchers in animal cognition. Chimpanzees’ cracking nuts with rocks and fishing termites with sticks are examples of flexible tool use (Biro et al., 2003; Lonsdorf, 2006), as a juvenile chimpanzee acquires such skills by observing its parent(s). Therefore, the learning happens at the level of the individual, rather than at the level of genus. Indeed, each instance of nut cracking or fishing termites can be differentiated even within the same context by the same chimpanzee. Unlike stereotyped tool use, flexible tool use does not share context-dependency and thus can occur across different contexts. We summarized the differences between stereotyped tool use and flexible tool use suggested by Hunt et al. in Table 1.

**TABLE 1:** Comparison of stereotyped tool use and flexible tool use based on Hunt et al. (2013)’s descriptions.

	Stereotyped tool use	Flexible tool use
Distribution	genus level	individual level
Development	phenotypic changes stemed from pre-existing non-tool use behaviors	observational learning
Variability	almost no variations within and between individuals	very different within and between individuals

Call (2013) further identified different types of flexible tool use from the perspective of problem-solving in terms of creativity and adaptivity.

• Solving novel problems with old solutions;
• Solving old problems with novel solutions;
• Solving novel problems with novel solutions.

Solutions may include utilizing one tool, selecting a tool from available options, manufacturing a novel tool, or using multiple tools sequentially.

In contrast to the above taxonomies, Wimpenny et al. (2009) categorized tool use based on the number of tools involved in a problem but overlooked the complexity of the decision process. Boesch (2013) categorized tool use based on four levels of increasing complexity though in some sense reminiscent of Wimpenny et al.‘s approach.

• *Simple tool use*: Using one tool, e.g., a chimpanzee uses a twig for fishing termites (Goodall, 1964). The animal only needs to understand the connection between itself and the reward *via* the tool, which is a first-order problem (Visalberghi and Fragaszy, 2006);
• *Combined tool use*: Using two tools simultaneously, e.g., a capuchin monkey uses a rock to pound a nut on a hard surface (Spagnoletti et al., 2011). The animal needs to consider both spatial relationships concurrently to connect itself with the reward, which is a second-order problem (Visalberghi and Fragaszy, 2006);
• *Sequential tool use*: Using multiple tools one after another, including using a tool for manufacturing another tool, e.g., a chimpanzee using multiple tools in sequence to break a bee hive, open honey chambers, and extract the honey. This behavior not only requires the animal to keep in mind multiple causal relationships sequentially and choose the correct sequence, but also imposes temporal delay for the reward;
• *Composite tool use*: Combining multiple tools to use as one tool, a tool use behavior yet to be discovered in animals and currently unique to humans.

### 3.2 Taxonomy of robot tool use

While the above taxonomies are based on animal studies, Tee et al. (2018, 2022) proposed a categorization based on default usages of tools in robotics, and identified three types of tools: category-I tools that “help to amplify/augment certain kinematic or dynamic aspects of functions that are already in an agents repertoire.” (p. 6439), category-II tools that are similar to category-I tools but “require actions different from what the agent would have performed, without the tool, to achieve these functions.” (p. 6439), and category-III “provide new functions that a human cannot perform without a tool.” (p. 6440) As an example, they categorized a vacuum cleaner as a Category-III tool because a robot cannot perform a cleaning task without this tool. However, a vacuum cleaner can be used as a rake to reach objects or as a hammer to hit objects in other contexts. In these contexts, the vacuum cleaner should be classified as category-I tools. Given that this categorization does not consider contexts of tool use, it will be challenging for a system following this categorization to perform flexible tool use, which is context-based.

**FIGURE 2:**
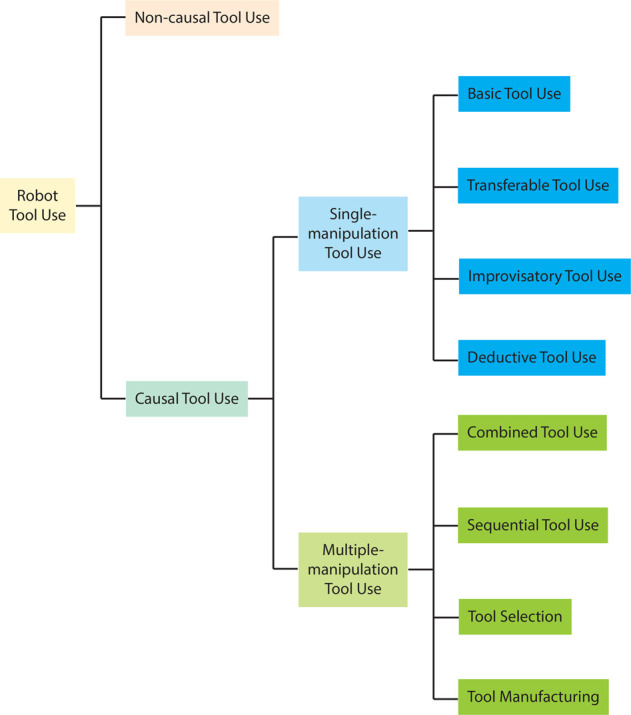
Tool use taxonomy.

Based on the taxonomies of animal tool use and the characteristics of robot tool use, we devise a taxonomy as shown in Figure 2. We categorize robot tool use into *non-causal tool use* and *causal tool use*, which are similar to stereotyped tool use and flexible tool use in animals, respectively. We changed the terminology for two reasons. Frist, we would like to emphasize the fundamental differences between the two types of tool use behaviors in robots regarding whether robots should understand required causal relations, which are elaborated in Section 4. Second, though we consider it necessary for a robot to understand required causal relations in order to achieve behaviors similar to flexible tool use, there is a lack of evidence showing the mechanism of flexible tool use in animals. Therefore, we would like to avoid claiming that flexible tool use in animals is causal-based, and such discussion is beyond the scope of this survey.

We further categorize causal tool use into *single-manipulation tool use* and *multiple-manipulation tool use* based on Boesch’s taxonomy. A single manipulation refers to being presented with a single tool and using the tool to perform one action (e.g., pushing, scooping) in order to achieve one goal, though a robot may observe the usage of multiple tools to learn a task. Multiple manipulations may involve one or any combinations of multiple tools, multiple actions, and goals consisting of multiple sub-goals.

Inspired by Call’s taxonomy, we categorize single-manipulation tool use into *basic tool use*, *transferable tool use*, *improvisatory tool use*, and *deductive tool use*. Basic tool use is the most basic form of tool use. In basic tool use, a robot uses a learned tool to solve a learned task, such as pushing a block, striking a xylophone, and cutting a cake. Unlike non-causal tool use that exclusively focuses on the actions, basic tool use focuses on the causal relations between actions and effects. Transferable tool use is a more complicated form of tool use, which aims to transfer learned tool use skills to other intra-category objects that share common form factors (e.g., using mugs of different shapes to pour liquid into different containers). Improvisatory tool use adds further complexity by generalizing learned tool use skills to novel inter-category objects. These objects are generally not designed to perform these tasks, such as using the handle of a screwdriver in place of a hammer to drive a nail. Deductive tool use concerns the problem of using a novel tool to solve a novel task. A robot will not be provided any prior knowledge about the tool or the task, nor given opportunities to learn about them from demonstrations. Instead, the robot should deduct the usage of a tool from its physical knowledge about the world.

We categorize multiple-manipulation tool use into *combined tool use*, *sequential tool use*, *tool selection*, and *tool manufacturing*. Combined tool use and sequential tool use are similar to the definitions as in Boesch’s taxonomy, though sequential tool use does not include constructing a new tool in our definition as it requires more sophisticated manipulation skills. Tool selection refers to the process of choosing the most appropriate tool among many options in order to complete a tool use task. Shumaker et al. (2011) defined tool manufacturing as “simply any structural modification of an object or an existing tool so that the object serves, or serves more effectively, as a tool.” This definition only includes modifying an existing object. We combine this definition with composite tool use in Boesch’s taxonomy, and re-define tool manufacturing as **the process of modifying or combining objects or existing tools, with or without the usage of other tools, to complete a tool use task, or to complete the task more efficiently**. Different from Shumaker et al. and Boesch’s definition, our definition also explicitly includes the possibility of utilizing other tools in the process of manufacturing.

We do not enforce a subdivision of multiple-manipulation tool use by difficulty, unlike a comparable category in Boesch’s taxonomy. Boesch was able to rank the categories in animal tool use because the types of tools leveraged by non-human animals are comparatively limited, and the manipulation skills in these animals are usually relatively simple. In robot tool use, the difficulty is dependent on the actual problem to solve, rather than the category that the problem belongs. For example, utilizing two tools in sequence may be simpler than creating a new tool that requires sophisticated manipulation skills. However, a problem that requires planning to use ten tools may be more challenging than a problem that requires a robot to combine two parts as a new tool.

As a summary, the criterion of the top-level classification is whether the context exhibit much variations across instances of tool use in the same task. As a result, robot tool use is classified into non-causal tool use and causal tool use. Causal tool use is further categorized into single-manipulation tool use and multiple-manipulation tool use based on the complexity of the task, which is quantified by the number of actions, goals, and tools. We categorize single-manipulation tool use into four sub-types. Three of the sub-types (i.e., basic tool use, transferable tool use, and improvisatory tool use) rely on prior experiences of tool use and the other (i.e., deductive tool use) only requires knowledge of physical rules or experiences of general manipulation. The three sub-types differ from each other in terms of object features. Single-manipulation tool use can be considered as building blocks of causal tool use, and multiple-manipulation tool use combines the building blocks in different ways. The different combinations form the sub-types of multiple-manipulation tool use, which are combined tool use, sequential tool use, tool selection, and tool manufacturing.

## 4 Required skills of robot tool use

Our definition of tool use has three important components: objects, goals or desired effects, and manipulations or actions to achieve the goals. These three components agree with the three ingredients of the affordance model defined by Montesano et al. (2008). The affordance model attempts to provide an operational definition of the concept of affordances, whose precise definition is still debatable (for a review, see Jamone et al., 2016; Zech et al., 2017). The concept was first introduced by Gibson (1979, p. 127) as what the environment “offers the animal, what it provides or furnishes, either for good or ill”. Despite the lack of consensus around its definition, the concept of affordances has facilitated much robotic research (Stoytchev, 2005b; Cakmak et al., 2007; Moldovan et al., 2012; 2013; Katz et al., 2014; Ruiz and Mayol-Cuevas, 2018; Lueddecke et al., 2019).

**FIGURE 3:**
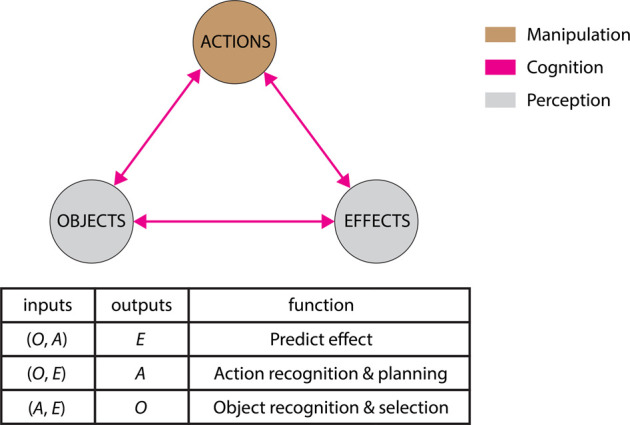
The modified affordance model in Montesano et al. (2008) © 2008, IEEE (reuse licence #5360371164789). Affordances as relations between (A)ctions, (O)bjects, and (E)ffects that can be used to address different purposes: predict the outcome of an action, plan actions to achieve a goal, or recognize objects or actions. We update the colors of the model and represent manipulation skills with brown, cognition skills with pink, and perception skills with grey.

The affordance model by Montesano et al. formulated affordance as three-way relations between objects, actions, and effects. Figure 3 shows our modified version of this affordance model with coloring. The coloring captures the three skills required for robot tool use as described in Section 1. While generating actions requires manipulation skills, perceiving the effects and the objects demands perception skills. Understanding the connections between the nodes in the model needs cognition skills. The key to robot tool use is to understand tool affordances.

**FIGURE 4:**
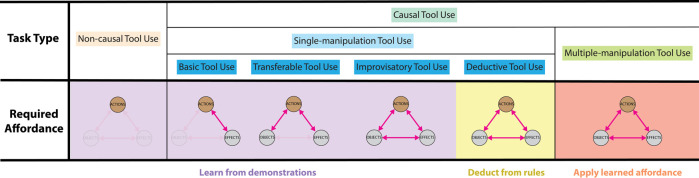
The different aspects of tool affordances need to be addressed in the subtype of tool use.

Each subtype of tool use in our taxonomy addresses different aspects of affordances and requires different skills, as shown in Figure 4. Non-causal tool use focuses on generating desired motions, which correspond to the action node in the affordance model. While the manipulation skills are similar to the ones in general manipulation tasks, tool use tasks require additional manipulation skills, such as updating a robot’s body schema when a tool is attached to its gripper. This type of tool use requires a robot to focus on the actions, though without the need to consider the objects, the effects, or the relations.

Causal tool use involves learning and applying tool affordances, which focuses on cognition skills. Single-manipulation tool use learns and reasons about affordances. Among the different types of single-manipulation tool use, basic tool use learns how to achieve desired effects with actions. With learned relations between actions and effects, transferable tool use learns the relations between tools and actions in order to adjust actions based on novel tools that share similar form factors with the learned tools. In addition to these two relations, improvisatory tool use requires a robot to understand what specific tool features cause the effects so that it can generalize learned skills to inter-category objects. As a result, improvisatory tool use requires a robot to learn the entire affordance model. These tool use tasks generalize tool use to novel objects by learning and inducing affordance from observations. In contrast, deductive tool use requires a robot to complete a tool use task without prior knowledge using unlearned tools. As a result, the robot has no information to induce affordances and should perform deductive reasoning from general physical rules. In short, basic tool use, transferable tool use, and improvisatory tool use requires the incremental learning of more relations from tool use demonstrations, while deductive tool use requires a robot to be able to infer rather than learn the relations from demonstrations of general manipulation tasks or physical rules provided directly.

The challenge for multiple-manipulation tool use is to apply the affordances effectively rather than learning the affordances. Tool manufacturing requires more sophisticated manipulation skills; sequential tool use and tool selection require higher cognition skills; tool manufacturing requires a high level of manipulation and cognition skills. We summarize the additional skills required in each sub-type in Table 2, and we elaborate more in Section 5.3.

**TABLE 2:** Additional skills required in multiple-manipulation tool use beyond single-manipulation tool use.

Additional manipulation skills		Additional cognition skills	
		Reasoning	Planning
Combined Tool Use	Coordinate the use of multiple tools	Choose appropriate parameters for each tool use	N/A
Sequential Tool Use	N/A	N/A	Plan the order of using multiple tools
Tool Selection	N/A	Choose appropriate tools among many available tools	N/A
Tool Manufacturing	Perform the actions of assembling different parts together as a tool, or modify the current tool	Choose appropriate parts to be assembled and decide where to attach each part, or decide the desired state of the unmodified tool	Planning the order of the manufacturing, which may include sequential tool use

As a summary, while non-causal tool use focuses on characterizing and replicating actions, causal tool use considers the relations between actions and other skills in addition. Under causal tool use category, single-manipulation tool use focuses on learning the skills and the relations while multiple-manipulation applies this knowledge to solve more complicated tasks and may require more sophisticated skills.

## 5 Robot tool use literature

In the previous sections, we present our definition of robot tool use drawing from the animal studies literature. Our definition highlights the three important skills in tool use, echoing the three-way relations between actions, objects, and effects in standard affordance models. We also describe a taxonomy of tool use with each type of tool use requiring different skills or relations.

In this section, we organize robot tool use literature based on our taxonomy. As different levels of assumptions can be made, we would like to point out that techniques that focus on the unique challenge in a higher-level tool use may not necessarily allow a robot to handle the challenges that belong to lower-level tool use. In each section, we focus on the techniques that can solve the unique challenge at each level of tool use. We would also like to emphasize that the purpose of robot tool use is not to mimic or model how animals use tools, but rather to allow robots to use tools.

### 5.1 Non-causal tool use

Non-causal tool use is the ability to use learned tools to solve learned tasks, without understanding the cause-and-effect relationship between the actions and the goals. The purpose of non-causal tool use is to duplicate or reproduce actions with limited variations. It could be achieved by programming a wide variety of tool use actions such as nut fastening in aircraft production that requires high precision (Pfeiffer et al., 2017), stub grinding and deburring with force control (Robertsson et al., 2006), handwriting that involves multi-contact manipulation (Kim et al., 2014), furniture polishing that uses an impedance model (Nagata et al., 2001), generating a collision-free polishing path (Takeuchi et al., 1993), accurately drawing a circle with a compass that involves complex contacts (Kutsuzawa et al., 2017), unfastening screws in collaborative tasks (Li et al., 2020), and pouring based on the volume of liquid (Rozo et al., 2013), or actions relevant to tool use such as grasping a knife resting on a cutting board that requires a high level of dexterity (Xue and Jia, 2020), or segmenting a surgical tool from the background while using it (Garcia-Peraza-Herrera et al., 2017; Su et al., 2018). The purpose of these approaches is to automate one process to facilitate human work. Therefore, the implementations are designed to be highly specific to the task.

However, given the wide range of tasks, it is impractical to program all tool use tasks. Being able to learn these tasks is desired. One approach is to treat tool use tasks the same way as general manipulation tasks and learn the actions accordingly. One of the classic algorithms of learning actions is dynamic movement primitives (DMP) (Schaal, 2006; Ijspeert et al., 2013). DMP leverages the concept of attractors from dynamical systems, and actions are represented as a set of linear differential equations. A more intuitive approach to understanding DMP is to visualize the equations as vector fields, where a trajectory is formed by following the vectors from a starting point to an end point. Each dimension may need to be learned separately and then coupled together. One advantage of DMP is that the shape of the trajectory can be distorted based on the starting point and the end point. DMP and its variations have been demonstrated with tool use tasks such as swinging a tennis racket (Ijspeert et al., 2002; Schaal, 2006), playing table tennis (Muelling et al., 2010), playing ball-in-a-cup (Kober et al., 2008), pouring liquid (Pastor et al., 2009), and whiteboard cleaning (Kormushev et al., 2011). Algorithms other than DMP have also been employed to represent action primitives, such as probabilistic movement primitives (Paraschos et al., 2013) and Fourier movement primitives (Kulak et al., 2020). Actions were also parameterized as minimal plans to facilitate action interpretation (Guha et al., 2013). To handle tasks that require high manipulation precision such as using chopsticks, model-free imitation learning was chosen (Ke et al., 2021). For tasks that do not require high precision of force or position control, indirect force controllers (Lutscher and Cheng, 2013) or a unified algorithm for dynamic object manipulation (Tsuji et al., 2015) can be considered. While these are methods designed to learn actions, general learning methods such as deep learning (Droniou et al., 2014; Byravan and Fox, 2017) and reinforcement learning (Peters and Schaal, 2006) were also used.

While these studies focus on learning actions, others focus on segmenting continuous actions into action primitives for tool use (Ramirez-Amaro et al., 2014a; Lioutikov et al., 2017). A similar line of research on general manipulation tasks is to recognize the tasks based on the classification of actions (Ramirez-Amaro et al., 2014b; 2015; Hu et al., 2014; Wölfel and Henrich, 2018; Shao et al., 2021; Koch et al., 2022). This approach attempts to ground action profiles to labels, either primitive labels or task labels, and do not relate actions to the effects on the objects being manipulated. For example, the whiteboard swiping action will be characterized as the translational movement of the eraser in this approach, rather than the words being erased, which is the effect. While grounding action profiles to labels is useful in some applications, it does not permit causal tool use.

The above studies treated tool use tasks in the same manner as general manipulation tasks. As a result, they cannot adjust actions based on how the tools are grasped since they do not have tool-related knowledge. In order to accommodate the tools attached to the end-effector, a robot needs to update its body schema to include the tools, or in other words, to calibrate the tool in the gripper. Prior studies focus on updating robot kinematics by considering the tip of a tool [e.g., Kemp and Edsinger (2006)], which is considered the primary contact point between the tool and the environment. Among these studies, some manipulated the tool with kinematic control (Stoytchev, 2003; Nabeshima et al., 2005), and others found it necessary to perform dynamic control (Kemp and Edsinger, 2006; Nabeshima et al., 2007; Jamone et al., 2013; Hoffmann et al., 2014; Karayiannidis et al., 2014). Despite these studies’ success, considering the tool’s tip only is insufficient for all tool use tasks. For example, it is insufficient to know where the tip of a mug is when it is used to pour liquid into another container. The mug needs to be tracked with multiple markers attached to it (Lee et al., 2008). Another example is joint tools such as a pair of scissors. In this scenario, a grounded relational representation of the entire tool is needed (Katz et al., 2008). Beyond tool calibration, other studies explored collision detection (Colgate et al., 1995) and obstacle avoidance (Lee and Song, 2021) with tools attached to the gripper, as well as robot motion planning to complete tool use tasks (Kobayashi and Hosoe, 2009; Holladay et al., 2019) or planning for the grasping of the tool (Lin and Sun, 2015; Chen et al., 2019; Raessa et al., 2019) in robot motion generation.

### 5.2 Causal tool use—Single-manipulation tool use

#### 5.2.1 Basic tool use

Though both basic tool use and non-causal tool use leverage learned tools to solve learned tasks, basic tool use can adjust actions based on the desired effects while non-causal tool use cannot. In other words, basic tool use requires robots to understand the causal relations between actions and effects. For example, a robot performing basic tool use is able to push an object further away given a target region that is further away, while a robot performing non-causal tool use will simply attempt to duplicate learned actions and does not adjust the actions based on the target region that is further away.

Sinapov and Stoytchev (2008) conducted an early study to explore the relation between actions and effects with motion babbling. They utilized six different tools (T-stick, L-stick, straight stick, L-hook, Y-hook, and an arrow-shaped tool) to relocate a puck with six pre-defined exploratory behaviors (i.e., push, pull, slide-left, slide-right, rotate-left, and rotate-right). For each tool, the robot learned the distribution of movement trajectories of the puck relative to its starting location. Forestier and Oudeyer (2016) employed an active version of Model Babbling to explore the distribution of manipulanda after tool use with two different sticks. These studies focus on the potential distribution of the location of manipulanda, rather than the one-to-one relationship between an action and its effect. Therefore, it is challenging to utilize tools to achieve desired effects with this method.

Other studies learned the one-to-one relation of an action and its effect, though in a quantitative manner. Okada et al. (2006) focused on verifying the effects as success or failure of tool use tasks such as pouring. Pastor et al. (2011) focused on predicting whether an object has been successfully struck by a pool cue or flipped using chopsticks. Studying the relation of an action and its effect in this manner is suitable if the state of the effects is discrete, but may not fit tool use tasks whose effects are continuous such as pushing an object 10 cm to its right.

Studies that focus on learning the one-to-one relation of an action and its effect in a qualitative way generally employed tasks that result in the relocation of manipulanda. Stoytchev (2005a, 2008) pre-defined eight pulling actions and recorded the effects of these actions with five different tools into an affordance table. As the actions were discretized, the effects can also be categorized in discretized space. In the evaluation, a robot needed to choose appropriate actions based on the affordance table in order to pull the manipulanda into a goal region, given one of the learned tools. Though Tikhanoff et al. (2013) also leveraged pre-defined actions, they allowed the actions to be parameterized with continuous variables, e.g., a randomly sampled pushing direction. Rather than keeping an affordance table, they used Least Square Support Vector Machines to regress the actions to the effects. Elliott et al. (2016) considered more types of push and pull. They also leveraged two regression techniques: linear regression and Gaussian process regression. Other than pulling and pushing tasks, Elliott and Cakmak (2018) explored cleaning tasks to relocate dirt. As the manipulanda are clusters of rigid bodies rather than a single rigid-body manipulandum, they represented the surface as a grid, and trained a pixel-level classifier to predict whether each pixel contains dirt after an action. The robots in the above studies explored tool use by themselves, pre-defined actions are necessary. In contrast, Liu et al. (2018) did not pre-define actions and took the method of imitation learning and learned with deep reinforcement learning.

These studies focus on pushing and pulling tasks. A common feature of these tasks is that the desired effect determines how a tool should contact a manipulandum. Other tool use tasks may permit multiple equally viable ways for a tool to make contact with a manipulandum to achieve the same effect. For example, pouring liquid from different orientations all result in the same effect of a container being filled. Claassens and Demiris (2011) conducted preliminary studies and termed such properties with affordance symmetries. Affordance symmetries are important because a robot will be able to generate different trajectories to complete a tool use task when the learned contact results in collision. However, few studies have explored this direction to our knowledge.

#### 5.2.2 Transferable tool use

Transferable tool use describes the ability to take tool use skills trained on an object to other intra-category objects defined by a common form factor. Therefore, the key to transferable tool use is to match the unlearned objects with learned objects.

We first present studies that concern specific types of tool use tasks. Most of these focused on relocation tasks such as pulling and pushing. Mar et al. (2017) and Nishide et al. (2011) leveraged self-organized maps to extract tool features to avoid the need to pre-defining the features. Takahashi et al. (2017) learned a model with a deep neural network that incorporated both grasping information and tool functions. Vogel et al. (2017) searched for the “sweet spot” of a novel baseball bat-like object when used to hit a baseball by sensing the force at the end-effector. Other studies considered pouring tasks. Kroemer et al. (2012) used a kernel-based approach to generalize learned action skills to novel objects. Brandi et al. (2014) performed warping to the point cloud of a learned container to match a novel container. Dong et al. (2019) adjusted pouring behavior by estimating the volume of liquid in the unknown containers. While these studies attempted to transfer tool use skills to novel tools, other studies explored how to act upon novel manipulanda. Gemici and Saxena (2014) sought to transfer cutting skills to food of varying physical properties such as hardness. Elliott et al. (2017) transferred learned surface cleaning actions to different surfaces, including surfaces of different sizes. Li et al. (2018) developed the Push-Net so that the system can push novel objects for re-positioning and re-orientation.

Though these studies demonstrated promising results on specific tool use tasks, it is unknown whether these algorithms could generalize to other types of tool use tasks. Therefore, other researchers investigated algorithms that transfer learned skills more broadly and demonstrated with multiple tool use tasks. Tee et al. (2018, 2022) matched the point cloud of unseen tools to the point cloud of the end-effector and arms of the robot to obtain the usage of the tools. The kPAM/kPAM 2.0 (Manuelli et al., 2019; Gao and Tedrake, 2021) used keypoints on the tools to represent shared global shape of the category of tools, and tool use skills were inferred from these keypoints. Stückler and Behnke (2014b); Stückler et al. (2016, 2013); Stückler and Behnke (2014a, 2015) considered the point cloud presentation of tools and performed deformable registration with different levels of resolution in order to match the overall shape of the tools.

The approach of these studies requires two steps: one to learn tool use skills as basic tool use, and one to learn the transfer process. Other studies merged the two steps and learned them in one step. Sinapov and Stoytchev (2007) incorporated the shape of the tool when learning tool use models for the pulling task. As a preliminary model, transfer was only demonstrated with tools of the same shape but different sizes. Gonçalves et al. (2014a,b) utilized a Bayesian network to learn how the actions and tool shapes influence the effects. The shape parameters include area, convexity, eccentricity, compactness, circleness, and squareness. Due to the large size of the network, it needed to be reduced to be able to train effectively. They validated their technique with pulling and pushing tasks. Dehban et al. (2016) took a similar approach but overcame the drawback of the need for a discretization of data. To be able to handle grasping, Mar et al. (2015) leveraged support vector machines to map geometric features between learned and novel tools for pulling.

There are pros and cons of these two approaches. Training everything in one step may be more convenient, but the feature space can be quite large and requires more data. Training in a modular way will make it easier to diagnose when the algorithm does not function as intended. It will also make it easier to modify or incorporate new features as the former requires the entire model to be retrained.

#### 5.2.3 Improvisatory tool use

Improvisatory tool use describes the ability to use tools in a creative way, which involves generalizing learned tool use skills from objects designed for the tasks to inter-category objects. These objects may not share common form factors with the canonical tools. Therefore, local features of the tools that lead to the desired effects should be identified.

While transferring tool use requires a robot to infer how actions are affected by novel tools given the relation between actions and effects, improvisatory tool use requires a robot to also understand what features of the tools caused the effects, which is the relation between tools and effects. In other words, improvisatory tool use calls for the learning of the full affordance model (Montesano et al., 2007; 2008).

In order to identify local features in unlearned tools, the function of a tool needs to be detected on a per-part basis. Insights can be gained from a related line of research that explores task-oriented grasping of novel objects. These studies made efforts to detect the functional part of a tool in different tasks so that the system can generate different grasping of the same object based on the task (Myers et al., 2015; Song et al., 2010; 2011b; a; Ek et al., 2010; Madry et al., 2012; Song et al., 2015; Murali et al., 2020; Kokic et al., 2017; Detry et al., 2017). Similar to these studies, studies that focus on part detection for tool use also leveraged geometric features. Schoeler and Wörgötter (2015) segmented the tools and used graphs to represent the relations between different tools parts. Nakamura and Nagai (2010) learned the full affordance model. They provided human static demonstrations without showing the course of actions, and detected local features with the Scale Invariant Feature Transform.

Given the functions of each tool part alone, a robot cannot realize improvisatory tool use since a robot have no knowledge about how to orient a tool. The robot needs to combine the tool parts information with tool use knowledge. Due to challenges in modeling grasping, Fitzgerald et al. (2019) achieved the goal with human-guided adaptation that gained information on how to improvise each tool from human demonstrators. To improvise tool use without the need of human demonstrations for each tool, Agostini et al. (2015) learned actions with a modified DMP and used a Repository of Objects and Attributes with Roles to detect potential usages of a tool. This method is based on matching the global shapes of tools. Though this method can perform some improvisatory tool use (e.g., utilizing a knife vertically for stirring in a way similar to using a spatula), the transfer is limited. Other studies considered both global and local features. Fang et al. (2020) and Xie et al. (2019) took 2D images as input and trained neural networks for improvisatory tool use. While these studies learned tool use skills and tool feature detection together, other attempts learned them in a modular manner; Jain and Inamura (2013) manually pre-defined local features, discretized actions for the pulling and pushing tasks, and trained a robot with a T-shaped tool. They claimed that the skills could be generalized to novel tools, though no demonstration was provided. The Keto framework (Qin et al., 2020) and the GIFT framework (Turpin et al., 2021) generated keypoints on the tools, such as grasping points and function points, based on local features. The robot then planned motion based on the keypoints. However, the keypoint approach may have difficulty on tasks where the tool contact point cannot be readily represented using only one point on the surface, such as a pencil sharpener whose contact is inside the object and the contact is more than a single point. Without using keypoints, Abelha and Guerin (2017), Gajewski et al. (2019), Abelha et al. (2016), and Guerin and Ferreira (2019) characterized the point cloud of a tool by approximating each of its segments with superquadrics and superparaboloids. They parametrized tool use with so-called p-tools, and demonstrated their technique with a wide range of tasks such as hammering and scooping in simulation or on a physical robot. Qin et al. (2021) developed an integrated system and learns basic tool use, rather than pre-define the tool usages as in other studies. The system can achieve both transferable tool use and improvisatory tool use by considering both global and local geometric features. While these studies utilized visual features to transfer tool use, Zhu et al. (2015) included both geometric and physical features such as mass.

#### 5.2.4 Deductive tool use

In deductive tool use, a robot should be able to utilize a novel tool to solve a task for which it has no prior knowledge. This is a very challenging task, and no current studies can perform deductive tool use to our knowledge. This type of tool use requires a robot to infer the entire affordance model, which is the relations between actions, effects, and tools, without tool use training samples as in improvisatory tool use.

### 5.3 Causal tool use—Multiple-manipulation tool use

Multiple-manipulation tool use involves many different types of tool use. Unlike single-manipulation tool use, the subtypes of multiple-manipulation tool use may not be interrelated. Compared with single-manipulation tool use, they may require more sophisticated manipulation skills and cognition skills such as planning, which are generally not needed in single tool use where only one tool use task is considered. In terms of tool knowledge, they usually require the full model of tool affordance knowledge.

#### 5.3.1 Combined tool use

Combined tool use refers to using multiple tools simultaneously, such as using a fork and a knife to cut a steak. No prior studies have demonstrated combined tool use to our knowledge. We identify two main challenges of combined tool use. The first challenge is at the cognition level. A robot should choose the appropriate parameters for each tool use, such as where to cut with the knife and where to stab the steak with the fork. The second challenge is at the manipulation level, which is how to coordinate the actions of each tool. It involves collision-free motion planning and adjusting the actions of one tool based on the other tool. For example, the force exerted on the fork to stabilize the steak is dependent on the course of the cutting action with the knife. Though generating collision-free motion planning may share similar techniques in multi-agent systems [for a review, see Rossi et al. (2018); Ismail et al. (2018); Rasheed et al. (2022)], how to choose appropriate parameters and how to coordinate tools are issues specific to tool use and may need to be handled differently from general manipulation tasks.

#### 5.3.2 Sequential tool use

Sequential tool use involves completing multiple tool use tasks in order. Yamazaki et al. (2010) designed an integrated system of daily assistive robots and applied it to the task of tidying and cleaning rooms. This system focused on failure detection and recovery, and manually defined the sequence of tasks to be completed. For a robot to be fully autonomous, the robot should be able to arrange appropriate orders and decide appropriate task parameters for each tool use task since the end state of a task is the start state of the next task.

This requirement falls under the topic of task and motion planning (TAMP) [for a review, see Garrett et al. (2021)]. As its name suggests, it integrates low-level motion planning which includes classic robotic manipulation techniques and high-level task planning which belongs to classic AI planning. Task planning aims to find an action skeleton to achieve a goal (e.g., pick up a pencil, use it to write, and put the pen down). Motion planning aims to find motion plans to execute in a robot (e.g., the joint states for each action). TAMP aims to find action parameters to connect task planning and motion planning (e.g., where to grasp the pencil to pick it up so the pencil can be used to write). TAMP currently has two main approaches to find action parameters: the sampling-based approach and the optimization-based approach. The sampling-based approach, which is used in the majority of TAMP studies, samples action parameters and tests the feasibility of the sampled combinations. Therefore, this approach may have difficulty when the solution space is relatively small since the probability of being able to sample the correct solution is small. In contrast, the optimization-based approach used optimization techniques such as logic-geometric programming (Toussaint et al., 2018) or sequential quadratic programming (Hadfield-Menell et al., 2016). It is able to handle problems with a small solution space more efficiently if the local optima can be handled properly. However, this approach generally requires a longer running time for tasks with many objects due to the increased dimension.

Sequential tool use has been demonstrated with optimization-based TAMP. Toussaint et al. (2018) enabled a robot in simulation to reach a tool that was initially out of reach with another tool in order to grab the target object. While they can handle tasks in a static environment, Migimatsu and Bohg (2020) improved the method with an object-centric approach to adapt to situations where objects were moved by other agents. Though this study was not demonstrated with sequential tool use, it has the potential to be applied to sequential tool use. Due to the current preliminary stage of tool use research, sequential tool use has not been demonstrated with a sampling-based approach to our knowledge.

In the above optimization-based TAMP approach, sequential tool use is only included as a demonstration to validate TAMP methods. Tool use, especially sequential tool use, usually includes multiple objects, which makes it challenging for the optimization-based approach. It is also challenging for the sampling-based approach since tool use tasks generally have a smaller solution space due to the additional constraints of tools. Therefore, alternative TAMP algorithms designed for sequential tool use may be needed due to the special requirements of tool use tasks compared with general manipulation tasks.

#### 5.3.3 Tool selection

Tool selection is the ability to choose the most appropriate tool among many options. In order to select the most appropriate object to be used as a ram to keep a door open, Levihn and Stilman (2014) identified four properties of a ram. In order to learn the properties, Wicaksono and Sammut (2016) demonstrated a robot with an instance of the pulling task, and the robot then performed experiments to generate hypotheses about what features are important. As an example of the hypotheses, “the hook (of the pulling tool, which is a tube) must be located on the same end of tube as the cube (manipulandum).” The hypotheses are expressed in Horn clauses so that the features are qualitative. To learn the features in a quantitative manner, Saito et al. (2018) and Brawer et al. (2020) learned the full model of tool affordances and performed tool selection.

#### 5.3.4 Tool manufacturing

Tool manufacturing is the ability to complete a tool use task by constructing a tool by combining available materials, modifying a tool, or both. As this process may involve combining different pieces, the manipulation skills required may be similar to the skills in robotic assembly. The peg-in-hole task, which is to insert a peg in a hole, is a standard task in robotic assembly. Researchers have explored methods to improve a robot’s performance, such as working with more complex parts with force-guided assembly (Dietrich et al., 2010) and increasing the speed of compliant manipulators (Bös et al., 2017) (For a review on robotic assembly with learning from demonstration, see Zhu and Hu, 2018). Beyond the peg-in-hole task, previous studies also considered the slide-in-the-groove assembly task (Peternel et al., 2015), and robot assembly that leveraged tool use such as hammering and wrenching (Gu et al., 2014).

Nair et al. (2019a,b) studied tool manufacturing by combining available parts. Their system was provided with examples of tool use, and selected appropriate parts as the grasping parts and function parts. The selection was made by comparing the similarity between the available parts and segmented parts of the demonstrated examples. The next step is to combine the parts selected with appropriate orientations. The system then performed tool use tasks to validate the assembled tool. Unlike robotic assembly, the manipulation skills required in these studies are relatively simple. It pre-defined three ways of attaching the different parts: pierce attachment, grasp attachment, and magnetic attachment. Sammut et al. (2015) designed a robot engineer to perform tool manufacturing. The robot engineer first identified important features of a tool use task, and then constructed the tool using 3D printing.

Tool manufacturing is a complicated task. The task settings of current studies reduce the difficulty of both manipulation and cognition skills. At the manipulation level, a robot may need to combine different parts with simple manipulation skills or leverage an external machine. While in animal or human tool use, the manipulation skills required in tool manufacturing are sophisticated and may even require using other tools. At the cognition level, the choice of available parts discretizes the solution space compared with the task whose solution space is continuous, such as a chimpanzee needing to make a hook to retrieve food. Tool manufacturing also requires tool affordance knowledge to identify important features of a tool to be assembled and requires planning skills to arrange the manipulation actions, especially when sequential tool use is needed.

### 5.4 Summary

In this section, we reviewed previous studies in robot tool use and summarized them in Supplementary Material. Many studies treated tool use tasks as general manipulation tasks and focused on programming or characterizing and duplicating the actions. The learning in these non-causal tool use tasks did not consider the objects nor the effects. As a result, robots learned tool use in this manner share similar characteristics as animals performing stereotyped tool use that the tool use demonstrate limited variations and challenge to adapt to different contexts.

As an emerging topic, relatively few studies have explored causal tool use. Early studies investigated the action-effect relations. Some studies focused on describing the effect space of an action or predicting the success or failure of an action, while other studies focused on learning how to adjust actions in order to achieve the desired effects. The latter mostly leveraged the pulling and/or the pushing tasks. To achieve transferable tool use, some studies focused on particular tasks and very few studies explored generic frameworks that may work with multiple tasks. Improvisatory tool use is even more challenging. To realize this type of tool use, some studies attempted to identify the functions of parts of a tool while others treated a tool in a holistic manner and detected key characters to represent the tool including the relations between different parts of tools. The former can generally achieve amazing results in tool use tasks whose local features are crucial to solve a task (e.g., the blade of a knife), and the latter enjoys advantages for tasks whose tools share common factors (e.g., mugs in different shapes). Though very few studies have attempted to handle both tasks. Different from these sub-types of single-manipulation tool use tasks, no previous studies have attempted deductive tool use.

Compared with single-manipulation tool use, fewer studies have addressed multiple-manipulation tool use. No previous studies reported that their systems can handle combined tool use; sequential tool use was usually treated as a regular manipulation task in TAMP; tool selection is most similar to single-manipulation tool use and gained most attention among multiple-manipulation tool use; only limited attempts have been made to address even simplified version of tool manufacturing.

## 6 Discussions

We defined robot tool use, provided a taxonomy of robot use, and identified the required skills in each category of tool use. As a summary, non-causal tool use focuses on the manipulation skills of using tools. Causal tool use focuses on learning or applying affordances. The sub-categories of single-manipulation tool use learn different parts of affordances. Basic tool use learns the actions-effects relation. Transferable tool use focuses on the tools-actions relation in addition to the actions-effects relation. Improvisatory tool use requires the knowledge of the full model. Deductive tool use generates affordances with general knowledge, rather than inducting the model from experiences or demonstrations. While single tool use relies on learning affordances, multiple-manipulation tool use leverages learned affordances and requires more sophisticated manipulation and/or higher-level cognition skills. In addition, we review literature on robot tool use. In this section, we discuss current or near future applications as well as future directions for robot tool use.

### 6.1 Current applications of robot tool use

**FIGURE 5:**
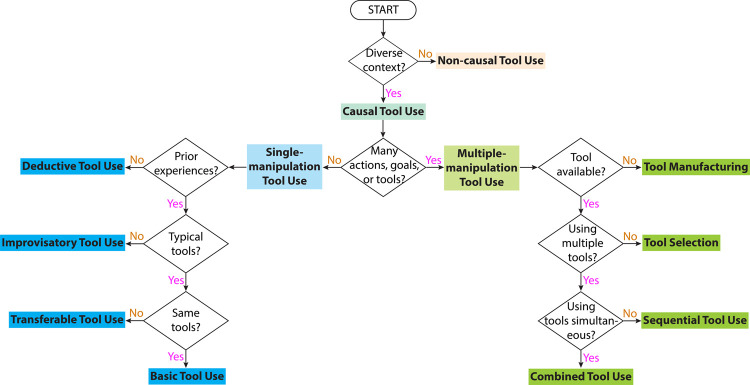
A cheat sheet for developing robot tool use applications.

The taxonomy can be used as a practical guideline for robot engineers when developing tool use applications. Robot engineers may start with the categorization criterion in the taxonomy since these are important features of robot tool use. This process also facilitates engineers to identify the sub-type of tool use involved in the application. We include a convenient cheat sheet for engineers as Figure 5. Upon the identification of the sub-type, our taxonomy also provides information regarding which skills should be focused on as summarized in Figure 4.

We would like to emphasize that the taxonomy was not designed to decide which type of tool use is superior, but to show the differences between the sub-types of tool use. For example, causal tool use may look more appealing than non-causal tool use as the actions can be generated more flexibly in different contexts. However, the former is more challenging to learn and the performance is comprised due to the limitations of current learning techniques. For industrial settings, robots on the assembly line may be required to complete the same task repeatedly. In this scenario, being more reliable may be more important than being more flexible. Moreover, the environment is designed for the robot and it is highly-controlled and being able to adapt to diverse context is less of a concern. As a result, non-causal tool use is sufficient. Conversely, household robots may face dynamic, complex, and stochastic environments. Such environments necessitate more flexible tool use skills. Therefore, causal tool use is required. In a nutshell, what kind of tool use to implement in a certain application is dependent on the need of the task and the limitations of current techniques.

We would also like to point out that the characteristics to classify the taxonomy may not be an exhaustive list for tool use in every scenario. For example, our taxonomy is designed to consider a robot solving a tool use task by itself. However, in practice, a robot may be required to solve a task jointly with a human collaborator. Considerations around safely handling tools, and doing so in a way that fosters effective collaboration are important, but beyond the scope of the taxonomy and this survey. Moreover, different applications may have significantly different requirements making it impractical to design a guideline that may apply to every conceivable application. Our taxonomy provides a basic guideline to design a robot to complete tool use tasks, and engineers should identify specific requirements of particular applications. Not to mention that the real-world environment is noisier which adds another layer of complexity.

### 6.2 Open challenges of future robot tool use

The study of tool use is still in the preliminary stages, and most studies aim to solve non-causal tool use and basic tool use. We identify the following open challenges in tool use.

1. *How can a robot learn the relations between tool-manipulanda contact poses and effects in transferable tool use?* There is a lack of studies on the relationship between tool-manipulanda contact poses and tool use effects. Most studies focus on the relation between trajectories and effects.
2. *How can an integrative system for improvisatory tool use handle a wide range of tasks?* While it is challenging to improvise tool use based on either local or global features, it is even more challenging to develop a system that can solve a wide range of tool use tasks. Such a system should decide whether local or global features should be considered, or choose features beyond geometric ones.
3. *How can a robot perform deductive tool use?* The challenge for deductive tool use is the lack of prior experiences. Current techniques for other sub-types of single-manipulation tool use performs inductive reasoning that learns affordances from experiences, and cannot be applied to deductive tool use.
4. *How can a robot perform multiple-manipulation tool use?* Multiple-manipulation tool use requires a robot to perform single-manipulation tool use. In addition, each sub-type in multiple-manipulation tool use requires more sophisticated manipulation skills or higher level cognition skills. Moreover, the additional skills differ among the sub-types of multiple-manipulation tool use.
5. *How can a robot learn the dynamics in causal tool use?* It is already challenging for current studies to consider tasks that can be achieved with only kinematic control. It will be even more challenging to incorporate dynamics as it adds additional dimensions to consider.
6. *Can we design a benchmark database for standard tool use tasks?* It is not trivial to design standard tool use tasks with a benchmark database of object models to facilitate comparisons between different algorithms. The requirements of the tasks should be detailed enough for precise replication. However, detailed requirements may lead to algorithms tailored for these tasks, and loss of generality. Moreover, it is challenging to select representative tools for improvisatory tool use as tools are expected to be used in creative manners. It is also impossible to include all possible tools for a given task due to the almost endless choices of physical objects that can be used as tools. It is also time-consuming to obtain the 3D model of an object.
7. *When and how can tool use knowledge be applied other areas in robotics?* Most studies that are relevant to tool use ignore the affordance model. For example, when learning robot grasping or robot handovers, a system typically observes how a human grasps a tool or hands over a tool, rather than inferring how a tool should be grasped or handed over based on the subsequent tasks. It is important for a system to be equipped with affordance knowledge since affordance causally determines how a tool should be grasped or handed over for to perform subsequent tool use tasks (Qin et al., 2022). However, not every study involving tool use requires a robot to learn the full affordance model, and it is important to identify which part of the model should be learned. Moreover, it also requires effort to connect tool use learning module with other modules, such as robot grasping and human-robot-collaboration tasks.
