# Supplementary material for: Robot tool use: A survey
Source: Front Robot AI. 2023 Jan 16;9:1009488. doi: 10.3389/frobt.2022.1009488 (PMC9885045; doi:10.3389/frobt.2022.1009488)
Supplement: Supplementary file 1 [file DataSheet1.PDF]

Supplementary Material

1 SUPPLEMENTARY DATA

We present five summary tables of the tool use studies described in this survey based on the tool use taxonomy.

ID	Non-causal Tool Use	General Learning?	Learning Specifics?	Tasks	Dynamics?	Robots	Note
1	Pfeiffer et al. (2017)	no	no	fastening bolts in aircraft production	yes	physical: HRP-2Kai humanoid robot	
2	Robertsson et al. (2006)	no	no	stub grinding, deburring	yes	physical: ABB Irb6400 industrial robot with an extended ABB S4CPlus control system	
3	Kim et al. (2014)	no	no	writing	yes	simulation: dynamic simulation	
4	Nagata et al. (2001)	no	no	polishing	yes	physical: an industrial robot JS-10	
5	Takeuchi et al. (1993)	no	no	polishing	yes	physical: an articulate-type polishing robot with 6 degrees of freedom	
6	Kutsuzawa et al. (2017)	no	no	using a compass to draw a circle	yes	physical: a manipulator with six degrees of freedom MOTOMAN-MH3F	
7	Li et al. (2020)	no	no	unfastening screws	yes	physical: KUKA LBR iiwa 14 R800	
8	Rozo et al. (2013)	no	no	pouring	yes	physical: RX60	
9	Xue and Jia (2020)	no	no	n/a	n/a	simulation: UR10 with Shadow Hand	grasping planning
10	Su et al. (2018)	no	no	n/a	yes	physical: the Raven II platform	nision-based surgical tool segmentation
11	Garcia-Peraza-Herrera et al. (2017)	no	no	n/a	n/a	no	surgery tool segmention from 2D image
12	Schaal (2006); Ijspeert et al. (2002)	yes	no	tennis swinging	yes	physical: a 30 DOF Sarcos Humanoid robot	action learning: Dynamic Movement Primitives (DMP)
13	Muelling et al. (2010)	yes	no	playing table tennis	yes	physical: a Barrett WAM arm	action learning: a Mixture of Motor Primitives (MoMP)
14	Kober et al. (2008)	yes	no	playing ball-in-a-cup	yes	simulation: an anthropomorphic SARCOS robot arm	action learning: an augmented version of the dynamic systems motor primitives
15	Pastor et al. (2009)	yes	no	pouring	yes	physical and simulation: demonstrated with a 7-DoF robot arm and reproduced the action with the the Sarcos Slave arm in simulation	action learning: Dynamic Movement Primitives (DMP)
16	Kormushev et al. (2011)	yes	no	surface cleaning	yes	physical: a 25-DOF Fujitsu HOAP-2 humanoid robot	action learning: an extension of Dynamic Movement Primitives (DMP)
17	Paraschos et al. (2013)	yes	no	n/a	yes	physical: a KUKA lightweight robot arm; simulation: 7-link simulated planar robot	action learning: probabilistic movement primitives (ProMP)
18	Kulak et al. (2020)	yes	no	polishing, drawing	yes	physical: a 7-DoF torque-controlled Panda robot	action learning: Fourier movement primitive (FMP)
19	Droniou et al. (2014)	yes	no	writing digits	yes	physical: iCub	action learning: deep neural network
20	Byravan and Fox (2017)	yes	no	n/a	no	no	action learning: deep neural network
21	Guha et al. (2013)	yes	no	slicing, joining, mashing, pouring, stirring	no	no	action learning: minimalist plan
22	Tsuji et al. (2015)	yes	no	turn over pancake with a spatula, for Back and-Forth Gliding Movements of a Spatula to Slide Objects on Top	yes	physical: Motoman-MH3F	action learning: a unified algorithm for generating a variety of movements from planning trajectories that satisfy such conditions
23	Lutscher and Cheng (2013)	yes	no	table wiping, drawing	yes	physical: KUKA LBR-IV lightweight arm	action learning: a generalized programming layer for indirect force controllers (IFCs)
24	Ke et al. (2021)	yes	no	using chopsticks	yes	physical: a custom-built 6-DOF robot	action learning: combating covariate shift in model-free imitation learning

25	Lioutikov et al. (2017)	yes	no	writing letters	yes	physical: a 7-DoF KUKA lightweight arme-quipped with a five finger DLR HIT Hand II as end effector	action segmentation: Probabilistic Segmentation (ProbS)
26	Ramirez-Amaro et al. (2014a,b, 2015)	yes	no	pouring, cutting, cutting, sprinkling	yes	physical: iCub	action segmentation: semantic reasoning
27	Hu et al. (2014)	yes	no	n/a	no	no	action recognition: soft labeling
28	Shao et al. (2021)	yes	no	pushing, pulling, lifting, pushing, pouring, hitting	yes	simulation: 7-DoF Franka Panda robot arm with a two-fingered Robotiq 2F-85 gripper with pybullet	action recognition: language grounding
31	Wölfel and Henrich (2018)	yes	no	cutting, scratching, drawing, ironing, inserting, pouring, scooping, screwing, drilling	yes	simulation: unknown	action recognition: combined vervalized effects
32	Koch et al. (2022)	yes	no	n/a	no	no	action recognition: a methods-time-measurement based approach
35	Stoytchev (2003)	yes	yes	n/a	no	simulation: a two-joint robot	updating body schema: the tip of a tool
36	Nabeshima et al. (2005)	yes	yes	n/a	no	physical: a customized robot	updating body schema: the tip of a tool
37	Nabeshima et al. (2007)	yes	yes	poking	yes	simulation: a 3-DOF robot	updating body schema: the tip of a tool
33	Kemp and Edsinger (2006)	yes	yes	n/a	yes	physical: Domo	updating body schema: the tip of a tool
34	Jamone et al. (2013)	yes	yes	n/a	yes	simulation: iCub dynamic simulator	updating body schema: the tip of a tool
38	Karayiannidis et al. (2014)	yes	yes	n/a	yes	physical: a 7-DOF velocity controlled manipulator controlled at 130 Hz with a wrist mounted ATI Mini45 6-axis force-torque sensor	updating body schema: the tip of a tool
39	Hoffmann et al. (2014)	yes	yes	drilling, drawing	yes	physical: the DARPA ARM robot for the drilling task, drawing task: an ST Robotics R17 5-DOF arm equipped with a linear gripper	updating body schema: the tip of a tool
40	Lee et al. (2008)	yes	yes	differet swings with a wooden sword, drinking from a coffee cup, different strokes with a tennis racket forehand stroke, different swings with a golf club	yes	physical: a humanoid robot; simulation: human skeleton	updating body schema: multiple points on tools
41	Katz et al. (2008)	yes	yes	n/a	yes	simulation: a simulated chain robot	updating body schema: the entire tool
42	Colgate et al. (1995)	yes	yes	n/a	n/a	no	collisions detection
43	Lee and Song (2021)	yes	yes	n/a	n/a	physical: Techman TM5-700 6-DOF manipulator	obstacle avoidance
44	Holladay et al. (2019)	yes	yes	hammer pulling, screw driving, wrench turning, knife cutting	yes	physical: ABB YuMi with custom printed finger	motion plannng
45	Kobayashi and Hosoe (2009)	yes	yes	using one object to move another another	no	simulation: 2D circle as a robot manipulator	motion planning
46	Raessa et al. (2019)	yes	yes	n/a	n/a	physical: a manufacturing cell with dual UR3 robots with a Robotiq F85 two finger grip on each arm	grasp planning
47	Chen et al. (2019)	yes	yes	vacuum sucking in order to move objects	n/a	physical and simulation: a dual-arm UR3 Robotiq F-85 parallel finger grippers	grasp planning
48	Lin and Sun (2015)	yes	yes	n/a	n/a	physical and simulation: a real Barrett hand equipped on a 6-DOF FANUC LR MATE 200iC robotic arm	grasp planning

Table S1: Summary of Non-causal Tool Use Studies. In this table, we summarize the following aspects: (general learning) whether the study involves any type of learning, including aspects in general manipulation; (learning specifics) whether the study learns any aspect that is specifically for tool use, rather than general manipulation; (tasks) the tool use tasks demonstrated in this study; (dynamics) whether the study considers the dynamics while using tools; (robots) the robot that is used to demonstrated the tool use tasks or relevant aspects in this study.

ID	Basic Tool Use	Actions	Effects	Tools	Actions ↔ Effects	Sensory Input	Dynamics?	Tasks	Robots
1	Sinapov and Stoytchev (2008)	six predefined exploratory behaviors: *push*, *pull*, *slide-left*, *slide-right*, *rotate-left*, *rotate-right*	displacement of a puck in 2D	labels	using motion babbling to incrementally learn an adaptive hierarchical representation of the range of the effects	2D image	no	pushing, pulling	simulation: CRS+ A251 arm
2	Forestier and Oudeyer (2016)	dynamic movement primitives (DMP)	displacement in 2D	unknown	an active version of Model Babbling, which is the modular active curiosity-drive model babbling (the MACOB architecture)	n/a	no	pulling with magnetic and hook	simulation: a 2D robot with three joints and a gripper
3	Okada et al. (2006)	predefined action primitives	boolean (success, failure)	point cloud	sensor-based behavior verification system	sequence of 6 Dof coordinates for actions, 2D image for effects	yes	water-pouring, dishwashing	physical: a life-sized humanoid robot HRP2-JSK
4	Pastor et al. (2011)	dynamic movement primitives (DMP)	boolean (success, failure)	unknown	reinforcement learning	pool stroke: information from the Hokuyo laser scanner; box flipping: information from a MicroStrain Intertia-Link attached in side the box	yes	pool stroke, box flipping using two chopsticks	physical: PR2
5	Stoytchev (2005, 2008)	Eight predefined actions: *extend arm (2 inches)*, *extend arm (5 inches)*, *slide left (2 inches)*, *slide left (5 inches)*, *slide right (2 inches)*, *slide right (5 inches)*, *contract arm (2 inches)*, *contract arm (5 inches)*	3D displacement of an object from two cameras	colors	using motion babbling to learn the affordance table	2D image	no	pushing, pulling	physical: a CRS+ A251 manipulator arm
6	Tikhanoff et al. (2013)	predefined push action with random chosen directions	displacement in 2D	tool tip position	Least Square Support Vector Machine (LSSVM)	3D image	yes	pushing, pulling	physical: iCub
7	Elliott et al. (2016)	ten predefined actions: *front center push*, *front side push right*, *front side push left*, *slide corner push right*, *slide corner push left*, *slide surface push right*, *slide surface push left*, *top pull*, *top side pull right*, *top side pull left*	3D displacement of an object	point cloud	multi-model regression	3D image	no	pushing, pulling	physical: PR2
8	Elliott and Cakmak (2018)	four predefined actions	boolean (success, failure) of each pixel		learning a predictive model of the task outcome from demonstrations	point cloud	no	cleaning (wiping or removing dirt)	physical: PR2; Fetch

9	Liu et al. (2018)	human actions from videos	object states	unknown	imitation learning based on video prediction with context translation and deep reinforcement learning	2D video	no	In simulation: pushing, sweeping, striking; Physical: sweeping, ladling almonds, pushing objects,	physical: a 7-DoF Sawyer robotmixure; simulation: the MuJoCo simulator
10	Claassens and Demiris (2011)	trajectories	3D point	3D point	affordance symmetry	information from an Opti-Track NaturalPoint motion capture system with 8 cameras	no	pouring, cutting	no

Table S2: Study Summary of Causal Tool Use — Single-Manipulation Tool Use — Basic Tool Use. In this table, we summarize the following aspects: (actions) the action representations; (effects) the effect representations; (tools) the tool representations; (Actions $\leftrightarrow$ Effects) how this study learns the relation between actions and effects; (sensory input) the type of sensory input; (dynamics) whether the study considers the dynamics while using tools; (tasks) the tool use tasks demonstrated in this study; (robots) the robot that is used to demonstrated the tool use tasks or relevant aspects in this study.

ID	Transferable Tool Use	Actions	Effects	Tools	Actions ↔ Effects	Tools ↔ Actions	Sensory Input	Dynamics?	Tasks	Robots
1	Mar et al. (2017)	eight predefined actions: displacing a tool 17 cm along eight different radial direction	object displacement	tool-pose descriptors	assumed	parallel Self-Organizing Maps (SOM) mapping	3D image	no	dragging	simulation: iCub
2	Nishide et al. (2011)	a series of robot's joint angles	a series of features extracted with Self-Organizing Maps (SOM)	dynamics learning module, which is multiple time-scales recurrent neural network (MTRNN)	clustering the calculated parametric bias (PB) value of tools	2D image	yes	pulling	physical: the humanoid robot HRP-2	
3	Takahashi et al. (2017)	a series of robot's joint angles	start and target images	2D image	motor babbling using deep neural network (DNN) and multiple time-scales recurrent neural network (MTRNN)	deep neural network (DNN)	2D image	yes	swinging, pulling	simulation: the humanoid robot ACTOROID
4	Vogel et al. (2017)	unknown (pre-programmed)	unknown	unknown	assumed	optimization of energy transfer	torque and jerk at end effector	yes	hitting tasks like stiking a ball with a bat	physical: DLR LWR III manipulator
5	Kroemer et al. (2012)	dynamic movement primitives (DMP)	unknown	non-parametric representation of surface structures	assumed	kernal logistic regression (KLR)	information from a time-of-flight camera	no	pouring	physical: a seven degrees-of-freedom Motoman robot arm, a seven degrees-of-freedom Motoman robot arm, and a five-fingered Gifu robot hand
6	Brandi et al. (2014)	probablistic motor primitives (ProMP)	unknown	point cloud	assumed	3D warping with predefined labels	3D image	no	pouring	physical: a dual-arm robot ; simulation: the Bullet physics engine [20] together with Fluids 2
7	Dong et al. (2019)	tilting angle of the container	estimated volume of the liquid	point cloud	assumed	estimating the volume of the container	3D image	yes	pouring	physical: dual-arm robot system

8	Gemici and Saxena (2014)	unknown (pre-programmed)	unknown	(manipulanda representation) six predefined physical properties: *hardness plasticity*, *elasticity*, *tensile strength*, *brittleness*, *adhesiveness*	assumed	Learning (Manipulanda Action): haptic learning with Dirichlet process and reinforcement learning	haptic inputs	yes	cutting	physical: PR2
9	Elliott et al. (2017)	extracted 2D clearning patterns	markers being removed	(manipulanda representation) size of a surface	assumed	Learning (Manipulanda Action): determine the repetition needed of the cleaning pattern which depends on the size of the surface	3D image	no	surface cleaning	physical: PR2
10	Li et al. (2018)	a tuple of start and end pixel on the image plane	displacement of an object, including rotation	(manipulanda representation) 2D pose and binary mask of an object	assumed	Push-Net	2D image	yes	pushing	physical and simulation: Fetch, Kinova MICO
11	Tee et al. (2018, 2022)	predefined actions: *pull back*, *push forward*, *push sideways*, *lift up*	object displacement	point cloud	assumed	matched the segmented unseen tools to segmented end-effector and arms of a robot with features extracted with 3D Orthogonal Profile Descriptors (OPD) technique	3D image	no	pulling, pushing, lifting	physical: a Olivia III robot; simulation: a planar three-DoF robot
12	Manuelli et al. (2019); Gao and Tedrake (2021)	the desired linear or angular velocity, or the desired force or torque of an oriented keypoint	unknown	keypoints of common form-factors	assumed	keypoint detection and rigid transformation	3D image	yes	whiteboard wiping, (proto-tool-use) peg-hole-insertion	
13	Stückler and Behnke (2014b); Stückler et al. (2016, 2013); Stückler and Behnke (2014a, 2015)	trajectories	unknown	point cloud	assumed	coherent point drift	3D image	yes	drawing, bottle opening, using a pair of tongs to grasp sausages, sweeping dust,watering plants	physical: cosero
14	Sinapov and Stoytchev (2007)	2D vectors	displacement of an object in 2D	tools in different frames	k-nearest and neighbor and decision tree		2D image	no	pulling	simulation: the open-source dynamic robot simulator BREVE

15	Gonçalves et al. (2014a,b); Dehban et al. (2016)	four pre-defined directional pushes: *left*, *right*, *pull closer*, *push away*	2D displacement of an object	pre-defined shape discriptors: *area*, *convex-ity*, *eccentricity*, *compactness*, *circleness*, *squareness*	probablistic causal model represented with Bayesian networks	2D im-age	no	pushing, pulling	physical and sim-ulation: iCub
16	Mar et al. (2015)	length and direction of a push	displacement of an object	pre-defined features (each include sub-features): *based on convex hull*, *based on thinning*, *moments*, *shape descriptors*, *from the angle sinature*, *domain transfor-mations from the distance to the centroid signature*	support vector machine classifiers	2D im-age	no	pulling	physical and sim-ulation: iCub

Table S3: Study Summary of Causal Tool Use — Single-Manipulation Tool Use — Transferable Tool Use. In this table, we summarize the following aspects: (actions) the action representations; (effects) the effect representations; (tools) the tool representations; (Actions $\leftrightarrow$ Effects) how this study learns the relation between actions and effects; (Tools $\leftrightarrow$ Actions) how this study learns the relation between tools and actions; (sensory input) the type of sensory input; (dynamics) whether the study considers the dynamics while using tools; (tasks) the tool use tasks demonstrated in this study; (robots) the robot that is used to demonstrated the tool use tasks or relevant aspects in this study.

ID	Improvisatory Tool Use	Actions	Effects	Tools	Actions ↔ Effects	Tools ↔ Actions	Tools ↔ Effects	Sensory Input	Dynamics?	Tasks	Robots	Note
1	Myers et al. (2015)	n/a	n/a	two approaches to learn local shape and geometry primitives: superixel based hierarchical matching pursuit (S-HMP); structured random forests (SRF)	n/a	n/a	n/a	3D image	no	cutting, scooping, containing, pounding	no	task-oriented grasping
2	Song et al. (2010, 2011b,a); Kroemer et al. (2012); Madry et al. (2012); Song et al. (2015)	n/a	n/a	two pre-defined features: *object class*; *object dimension*, *convexity*, *eccentricity*	n/a	n/a	n/a	3D image	no	pouring, tool-use	simulation: a 20-DoF human hand model, a 7-DoF Schunk Dexterous hand model, and an Armar 11 DoF hand	task-oriented grasping
3	Murali et al. (2020)	n/a	n/a	PointNet++ architecture [citation153 42] to represent point cloud and grasp poses; Sematic hierarchy of objects (WordNet)	n/a	n/a	n/a	3D image	no	mixing, sauting with a pan, can opening, spraying	physical and simulation: Sawyer Robot	task-oriented grasping
4	Kokic et al. (2017)	n/a	labels	point cloud	n/a	n/a	n/a	3D image	no	cutting, poking, pounding, pouring	simulation: a Schunk-SDH hand mounted on a KUKA KR5 sixx 850 manipulator	task-oriented grasping;
5	Detry et al. (2017)	n/a	labels	depth image	n/a	n/a	n/a	3D image	no	pouring	no	task-oriented grasping
6	Schoeler and Wörgötter (2015)	n/a	labels	graph representation pf segmented point cloud	n/a	n/a	support vector machines	3D image	no	sieving, cutting, containing, poking, hitting	no	tool part detection

7	Nakamura and Nagai (2010)	contact poses (grasping and tool-manipulandum contact poses)	four predefined features: *color change*, *contour change*, *Barycentric position change*, *change in the number of the work object*	scale invariant feature transform (SIFT) to represent local features	Bayesian networks			unknown	no	cutting, coloring, deformation, transfer, bonding, bonding with coloring	no	tool part detection
8	Fitzgerald et al. (2019)	linear and/or rotational transform model of the end-effector trajectory	n/a	n/a	assumed	inferred from human demonstrations for each tool	provided	unknown	no	sweeping, hooking, hammering	physical: a 7-DOF Jaco2 arm equipped with a Robotiq 85 gripper	
9	Agostini et al. (2015)	semantic event chains (SECs) enriched with object and trajectory information	predicates	segmented image	provided planning operator (PO)	database: a repository of objects and attributes with roles (ROAR)		unknown	yes	cutting, stirring	physical: the KUKA arm platform	
10	Fang et al. (2020)	predefined gripper trajectories	labels	point cloud	assumed	deep neural networks		3D image	no	sweeping, hammering	physical and simultation: a 7-DoF Rethink Robotics Sawyer Arm with a parallel jaw gripper	
11	Xie et al. (2019)	trajectories of end-effectors	position changes of objects in pixels	2D image	deep neural networks			2D image	no	sweeping, wiping, hooking	physical: a Sawyer robot	
12	Jain and Inamura (2013)	five predefined actions: *contract arm*, *slide arm left*, *pull diagonally-1*, *slide arm right*, *pull diagonally-2*	object displacement	three pre-defined features: *horizontal part*, *vertical part*, *corner*	Bayesian networks			2D image	yes	pushing, pulling	no	
13	Qin et al. (2020)	trajectories	labels	keypoints based on local features	assumed	a framework of keypoint representations for tool manipulation (KETO)		3D image	yes	hammering, pushing, reaching	simulation: pybullet	
14	Turpin et al. (2021)	contact poses (grasping and tool-manipulandum contact poses)	labels	keypoints based on local features	assumed	Generilizable Interaction-aware Functional Tool affordances (GIFT)		3D image	no	hooking, reaching, hammering	simulation: a Sawyer robot arm	

15	Abelha and Guerin (2017); Gajewski et al. (2019); Abelha et al. (2016); Guerin and Ferreira (2019)	predefined action profiles	labels	segmented point cloud approximated with superquadrics and superparaboloids	assumed	p-tools	3D image	no	rolling dough, cutting lasagne, hammering nail, lifting pancake, tenderising meat, piercing a potato skin, scooping	simulation: no actual robot	
16	Zhu et al. (2015)	trajectories	object status change	point cloud, mass, volume	ranking function		3D image	yes	chopping wood, shovelling dirt, painting wall	no	
17	Qin et al. (2021)	trajectories segmented with exponential representations of transformations	object displacement for relocation tasks; other properties for other tasks	point cloud	by classifying task type given demonstrations	2-steps substitution algorithm	3D image	no	knocking, stirring, pushing, scooping, cutting, writing, screw-driving	physical and simulation: Baxter, University Robotics UR5e, Kuka youBot	

Table S4: Study Summary of Causal Tool Use — Single-Manipulation Tool Use — Improvisatory Tool Use. In this table, we summarize the following aspects: (actions) the action representations; (effects) the effect representations; (tools) the tool representations; (Actions $\leftrightarrow$ Effects) how this study learns the relation between actions and effects; (Tools $\leftrightarrow$ Actions) how this study learns the relation between tools and actions; (Tools $\leftrightarrow$ Effects) how this study learns the relation between tools and effects; (sensory input) the type of sensory input; (dynamics) whether the study considers the dynamics while using tools; (tasks) the tool use tasks demonstrated in this study; (robots) the robot that is used to demonstrated the tool use tasks or relevant aspects in this study.

ID	Multiple-manipulation Tool Use	Category	Affordance	Manipulation	Cognition Reasoning	Cognition: Planning	Tasks	Robots	Note
1	Yamazaki et al. (2010)	Sequential Tool Use	assumed	no	no	pre-defined sequence; focus on failure detection and recovery	sweeping a floor	physical: ART daily assistive robot	
2	Toussaint et al. (2018)	Sequential Tool Use	provided in the form of equations	no	no	optimization-based task and motion planning (logic-geometric programming, LGP)	throwing, hitting, hitslide, hitslidesit, pushing	simulation: 14DOF humanoid with two arms	
3	Migimatsu and Bohg (2020)	Sequential Tool Use	provided in the form of equations	no	no	optimization-based task and motion planning (object centric logic-geometric programming, LGP)	n/a	physical and simulation: a 7-dof Franka Panda fitted with a Robotiq 2F-85 gripper	
4	Qin et al. (2022)	Sequential Tool Use	learned with TRI-STAR (Qin et al., 2021)	no	no	sampling-based task and motion planning (TAAMP)	pushing, pulling	physical: a Kuka youBot robot arm; simulation: a Kuka iiwa robot arm	
5	Levihn and Stilman (2014)	Tool Selection	prespecified tool features; physical laws	no	no	no	using a lever, or other objects to keep a door open	simulation: the dynamic simulator DART	
6	Wicaksono and Sammut (2016)	Tool Selection	formed hypothesis based on observations of tool use, and generated structural similarity score to select tools	no	no	no	pulling	physical and simulation: Baxter	
7	Saito et al. (2018)	Tool Selection	learned tool use with deep learning	no	no	no	pulling, sliding	physical: the NEXTAGE humanoid robot	
8	Brawer et al. (2020)	Tool Selection	learned tool use as a structural causal model (SCM) 8 3	no	no	no	pushing, pulling	physical: Baxter	
9	Dietrich et al. (2010)	Tool Manufacturing	n/a	the analysis of contact models for assembly	no	no	(pseudo-tool use) peg-in-hole with complex parts	physical: the parallel robot Hexa	robot assembly
10	Bös et al. (2017)	Tool Manufacturing	n/a	to increase the achievable speed of compliant manipulators with interatively learned and temporally scaled force control	no	no	(pseudo-tool use) peg-in-hole	physical: an ABB YuMi	robot assembly

12	Peternel et al. (2015)	Tool Manufacturing	n/a	impedance control interface for human-in-the-loop approach	no	no	sliding a bolt fitting inside a groove in order to mount two parts together	physical: Kuka Light Weight Robot, HapticMaster robot	robot assembly
13	Gu et al. (2014)	Tool Manufacturing	learning the relationship of actions and effects with a Portable Assembly Demonatration (PAD) system	no	no	no	screwing, hammering, wrenching	no	robot assembly
14	Nair et al. (2019a,b)	Tool Manufacturing	tool substitution algorithm from Abelha et al. (2016) with an extra step of tool validation phase	pre-determined method to attach parts together, including pierce attachment, grasp attachment and magnetic attachment	using computed scores to determine which parts to choose, and where to connect the parts	not needed as it only involves connecting two parts as a tool	hitting, containing or scooping, screwing, flipping, squeegeeing	physical: a 7-DOF robot arm	
15	Sammut et al. (2015)	Tool Manufacturing	learn affordances by forming hypothesis about important features of tools (Wicaksono, 2020)	constructed the tool with 3D printing	to determine the functional specification and to convert the specification into a design	not needed as the construction is by 3D printing	no evaluation yet	no	

Table S5: Study Summary of Causal Tool Use — Multiple-manipulation Tool Use. In this table, we summarize the following aspects: (category) the sub-category of this task in multiple-manipulation tool use; (affordance) how the affordance is learned in this study; (manipulation) extra manipulation skills needed compared with single-manipulation tool use; (cognition: reasoning) extra reasoning skills needed compared with single-manipulation tool use; (cognition: planning) extra planning skills needed compared with single-manipulation tool use; (tasks) the tool use tasks demonstrated in this study; (robots) the robot that is used to demonstrate the tool use tasks or relevant aspects in this study.

REFERENCES

Abelha, P. and Guerin, F. (2017). Learning how a tool affords by simulating 3d models from the web. In *2017 IEEE/RSJ International Conference on Intelligent Robots and Systems (IROS)* (IEEE), 4923–4929

Abelha, P., Guerin, F., and Schoeler, M. (2016). A model-based approach to finding substitute tools in 3d vision data. In *2016 IEEE International Conference on Robotics and Automation (ICRA)* (IEEE), 2471–2478

Agostini, A., Aein, M. J., Szedmak, S., Aksoy, E. E., Piater, J., and Würgütter, F. (2015). Using structural bootstrapping for object substitution in robotic executions of human-like manipulation tasks. In *2015 IEEE/RSJ International Conference on Intelligent Robots and Systems (IROS)* (IEEE), 6479–6486

Bös, J., Wahrburg, A., and Listmann, K. D. (2017). Iteratively learned and temporally scaled force control with application to robotic assembly in unstructured environments. In *2017 IEEE International Conference on Robotics and Automation (ICRA)* (IEEE), 3000–3007

Brandi, S., Kroemer, O., and Peters, J. (2014). Generalizing pouring actions between objects using warped parameters. In *2014 IEEE-RAS International Conference on Humanoid Robots* (IEEE), 616–621

Brawer, J., Qin, M., and Scassellati, B. (2020). A causal approach to tool affordance learning. In *2020 IEEE/RSJ International Conference on Intelligent Robots and Systems (IROS)* (IEEE), 8394–8399

Byravan, A. and Fox, D. (2017). Se3-nets: Learning rigid body motion using deep neural networks. In *2017 IEEE International Conference on Robotics and Automation (ICRA)* (IEEE), 173–180

Chen, H., Wan, W., and Harada, K. (2019). Combined task and motion planning for a dual-arm robot to use a suction cup tool. In *2019 IEEE-RAS 19th International Conference on Humanoid Robots (Humanoids)* (IEEE), 446–452

Claassens, J. and Demiris, Y. (2011). Generalising human demonstration data by identifying affordance symmetries in object interaction trajectories. In *2011 IEEE/RSJ International Conference on Intelligent Robots and Systems* (IEEE), 1980–1985

Colgate, J. E., Stanley, M. C., and Brown, J. M. (1995). Issues in the haptic display of tool use. In *Proceedings 1995 IEEE/RSJ International Conference on Intelligent Robots and Systems. Human Robot Interaction and Cooperative Robots* (IEEE), vol. 3, 140–145

Dehban, A., Jamone, L., Kampff, A. R., and Santos-Victor, J. (2016). Denoising auto-encoders for learning of objects and tools affordances in continuous space. In *2016 IEEE International Conference on Robotics and Automation (ICRA)* (IEEE), 4866–4871

Detry, R., Papon, J., and Matthies, L. (2017). Task-oriented grasping with semantic and geometric scene understanding. In *2017 IEEE/RSJ International Conference on Intelligent Robots and Systems (IROS)* (IEEE), 3266–3273

Dietrich, F., Buchholz, D., Wobbe, F., Sowinski, F., Raatz, A., Schumacher, W., et al. (2010). On contact models for assembly tasks: Experimental investigation beyond the peg-in-hole problem on the example of force-torque maps. In *2010 IEEE/RSJ international conference on intelligent robots and systems* (IEEE), 2313–2318

Dong, C., Takizawa, M., Kudoh, S., and Suehiro, T. (2019). Precision pouring into unknown containers by service robots. In *2019 IEEE/RSJ International Conference on Intelligent Robots and Systems (IROS)* (IEEE), 5875–5882

Droniou, A., Ivaldi, S., and Sigaud, O. (2014). Learning a repertoire of actions with deep neural networks. In *4th International Conference on Development and Learning and on Epigenetic Robotics* (IEEE), 229–234

Elliott, S. and Cakmak, M. (2018). Robotic cleaning through dirt rearrangement planning with learned transition models. In *2018 IEEE International Conference on Robotics and Automation (ICRA)* (IEEE), 1623–1630

Elliott, S., Valente, M., and Cakmak, M. (2016). Making objects graspable in confined environments through push and pull manipulation with a tool. In *2016 IEEE international conference on robotics and automation (ICRA)* (IEEE), 4851–4858

Elliott, S., Xu, Z., and Cakmak, M. (2017). Learning generalizable surface cleaning actions from demonstration. In *2017 26th IEEE International Symposium on Robot and Human Interactive Communication (RO-MAN)* (IEEE), 993–999

Fang, K., Zhu, Y., Garg, A., Kurenkov, A., Mehta, V., Fei-Fei, L., et al. (2020). Learning task-oriented grasping for tool manipulation from simulated self-supervision. *The International Journal of Robotics Research* 39, 202–216

Fitzgerald, T., Short, E., Goel, A., and Thomaz, A. (2019). Human-guided trajectory adaptation for tool transfer. In *Proceedings of the 18th International Conference on Autonomous Agents and MultiAgent Systems*. 1350–1358

Forestier, S. and Oudeyer, P.-Y. (2016). Modular active curiosity-driven discovery of tool use. In *2016 IEEE/RSJ International Conference on Intelligent Robots and Systems (IROS)* (IEEE), 3965–3972

Gajewski, P., Ferreira, P., Bartels, G., Wang, C., Guerin, F., Indurkhya, B., et al. (2019). Adapting everyday manipulation skills to varied scenarios. In *2019 International Conference on Robotics and Automation (ICRA)* (IEEE), 1345–1351

Gao, W. and Tedrake, R. (2021). kpam 2.0: Feedback control for category-level robotic manipulation. *IEEE Robotics and Automation Letters* 6, 2962–2969

Garcia-Peraza-Herrera, L. C., Li, W., Fidon, L., Gruijthuijsen, C., Devreker, A., Attilakos, G., et al. (2017). Toolnet: holistically-nested real-time segmentation of robotic surgical tools. In *2017 IEEE/RSJ International Conference on Intelligent Robots and Systems (IROS)* (IEEE), 5717–5722

Gemici, M. C. and Saxena, A. (2014). Learning haptic representation for manipulating deformable food objects. In *2014 IEEE/RSJ International Conference on Intelligent Robots and Systems* (IEEE), 638–645

Gonçalves, A., Abrantes, J., Saponaro, G., Jamone, L., and Bernardino, A. (2014a). Learning intermediate object affordances: Towards the development of a tool concept. In *4th International Conference on Development and Learning and on Epigenetic Robotics* (IEEE), 482–488

Gonçalves, A., Saponaro, G., Jamone, L., and Bernardino, A. (2014b). Learning visual affordances of objects and tools through autonomous robot exploration. In *2014 IEEE International Conference on Autonomous Robot Systems and Competitions (ICARSC)* (IEEE), 128–133

Gu, Y., Sheng, W., and Ou, Y. (2014). Automated assembly skill acquisition through human demonstration. In *2014 IEEE international conference on robotics and automation (ICRA)* (IEEE), 6313–6318

Guerin, F. and Ferreira, P. (2019). Robot manipulation in open environments: New perspectives. *IEEE transactions on cognitive and developmental systems* 12, 669–675

Guha, A., Yang, Y., Fermu, C., Aloimonos, Y., et al. (2013). Minimalist plans for interpreting manipulation actions. In *2013 IEEE/RSJ International Conference on Intelligent Robots and Systems* (IEEE), 5908–5914

Hoffmann, H., Chen, Z., Earl, D., Mitchell, D., Salemi, B., and Sinapov, J. (2014). Adaptive robotic tool use under variable grasps. *Robotics and Autonomous Systems* 62, 833–846

Holladay, R., Lozano-Pérez, T., and Rodriguez, A. (2019). Force-and-motion constrained planning for tool use. In *2019 IEEE/RSJ International Conference on Intelligent Robots and Systems (IROS)* (IEEE), 7409–7416

Hu, N., Lou, Z., Englebienne, G., Kröse, B. J., et al. (2014). Learning to recognize human activities from soft labeled data. In *Robotics: Science and Systems*

Ijspeert, A. J., Nakanishi, J., and Schaal, S. (2002). Movement imitation with nonlinear dynamical systems in humanoid robots. In *Proceedings 2002 IEEE International Conference on Robotics and Automation (Cat. No. 02CH37292)* (IEEE), vol. 2, 1398–1403

Jain, R. and Inamura, T. (2013). Bayesian learning of tool affordances based on generalization of functional feature to estimate effects of unseen tools. *Artificial Life and Robotics* 18, 95–103

Jamone, L., Damas, B., Santos-Victor, J., and Takanishi, A. (2013). Online learning of humanoid robot kinematics under switching tools contexts. In *2013 IEEE International Conference on Robotics and Automation* (IEEE), 4811–4817

Karayiannidis, Y., Smith, C., Vina, F. E., and Kragic, D. (2014). Online contact point estimation for uncalibrated tool use. In *2014 IEEE International Conference on Robotics and Automation (ICRA)* (IEEE), 2488–2494

Katz, D., Pyuro, Y., and Brock, O. (2008). Learning to manipulate articulated objects in unstructured environments using a grounded relational representation. In *In Robotics: Science and Systems* (Citeseer)

Ke, L., Wang, J., Bhattacharjee, T., Boots, B., and Srinivasa, S. (2021). Grasping with chopsticks: Combating covariate shift in model-free imitation learning for fine manipulation. In *2021 IEEE International Conference on Robotics and Automation (ICRA)* (IEEE), 6185–6191

Kemp, C. C. and Edsinger, A. (2006). Robot manipulation of human tools: Autonomous detection and control of task relevant features. In *Proc. of the Fifth Intl. Conference on Development and Learning*. vol. 42

Kim, S.-K., Jo, J., Oh, Y., Oh, S.-R., Srinivasa, S., and Likhachev, M. (2014). Robotic handwriting: multi-contact manipulation based on reactional internal contact hypothesis. In *2014 IEEE/RSJ International Conference on Intelligent Robots and Systems* (IEEE), 877–884

Kobayashi, Y. and Hosoe, S. (2009). Planning-space shift learning: Variable-space motion planning toward flexible extension of body schema. In *2009 IEEE/RSJ International Conference on Intelligent Robots and Systems* (IEEE), 3107–3114

Kober, J., Mohler, B., and Peters, J. (2008). Learning perceptual coupling for motor primitives. In *2008 IEEE/RSJ International Conference on Intelligent Robots and Systems* (IEEE), 834–839

Koch, J., Büsch, L., Gomse, M., and Schüppstuhl, T. (2022). A methods-time-measurement based approach to enable action recognition for multi-variant assembly in human-robot collaboration. *Procedia CIRP* 106, 233–238

Kokic, M., Stork, J. A., Haustein, J. A., and Kragic, D. (2017). Affordance detection for task-specific grasping using deep learning. In *2017 IEEE-RAS 17th International Conference on Humanoid Robotics (Humanoids)* (IEEE), 91–98

Kormushev, P., Nenchev, D. N., Calinon, S., and Caldwell, D. G. (2011). Upper-body kinesthetic teaching of a free-standing humanoid robot. In *2011 IEEE International Conference on Robotics and Automation* (IEEE), 3970–3975

Kroemer, O., Ugur, E., Oztop, E., and Peters, J. (2012). A kernel-based approach to direct action perception. In *2012 IEEE international Conference on Robotics and Automation* (IEEE), 2605–2610

Kulak, T., Silvério, J., and Calinon, S. (2020). Fourier movement primitives: an approach for learning rhythmic robot skills from demonstrations. In *Robotics: Science and Systems*

Kutsuzawa, K., Sakaino, S., and Tsuji, T. (2017). A control system for a tool use robot: Drawing a circle by educing functions of a compass. *Journal of Robotics and Mechatronics* 29, 395–405

Lee, D., Kunori, H., and Nakamura, Y. (2008). Association of whole body motion from tool knowledge for humanoid robots. In *2008 IEEE/RSJ International Conference on Intelligent Robots and Systems* (IEEE), 2867–2874

Lee, Y.-H. and Song, K.-T. (2021). Real-time obstacle avoidance with a virtual torque approach for a robotic tool in the end effector. In *2021 IEEE International Conference on Robotics and Automation (ICRA)* (IEEE), 8436–8442

Levihn, M. and Stilman, M. (2014). Using environment objects as tools: Unconventional door opening. In *2014 IEEE/RSJ International Conference on Intelligent Robots and Systems* (IEEE), 2502–2508

Li, J. K., Lee, W. S., and Hsu, D. (2018). Push-net: Deep planar pushing for objects with unknown physical properties. In *Robotics: Science and Systems*. vol. 14, 1–9

Li, R., Pham, D. T., Huang, J., Tan, Y., Qu, M., Wang, Y., et al. (2020). Unfastening of hexagonal headed screws by a collaborative robot. *IEEE Transactions on Automation Science and Engineering* 17, 1455–1468

Lin, Y. and Sun, Y. (2015). Robot grasp planning based on demonstrated grasp strategies. *The International Journal of Robotics Research* 34, 26–42

Lioutikov, R., Neumann, G., Maeda, G., and Peters, J. (2017). Learning movement primitive libraries through probabilistic segmentation. *The International Journal of Robotics Research* 36, 879–894

Liu, Y., Gupta, A., Abbeel, P., and Levine, S. (2018). Imitation from observation: Learning to imitate behaviors from raw video via context translation. In *2018 IEEE International Conference on Robotics and Automation (ICRA)* (IEEE), 1118–1125

Lutscher, E. and Cheng, G. (2013). A practical approach to generalized hierarchical task specification for indirect force controlled robots. In *2013 IEEE/RSJ International Conference on Intelligent Robots and Systems* (IEEE), 1854–1859

Madry, M., Song, D., Ek, C. H., and Kragic, D. (2012). "robot bring me something to drink from": object representation for transferring task specific grasps. In *ICRA Workshop on semantic perception, mapping and exploration*. 1–6

Manuelli, L., Gao, W., Florence, P., and Tedrake, R. (2019). kpam: Keypoint affordances for category-level robotic manipulation. In *The International Symposium of Robotics Research* (Springer), 132–157

Mar, T., Tikhanoff, V., Metta, G., and Natale, L. (2015). Self-supervised learning of grasp dependent tool affordances on the icub humanoid robot. In *2015 IEEE International Conference on Robotics and Automation (ICRA)* (IEEE), 3200–3206

Mar, T., Tikhanoff, V., Metta, G., and Natale, L. (2017). Self-supervised learning of tool affordances from 3d tool representation through parallel som mapping. In *2017 IEEE International Conference on Robotics and Automation (ICRA)* (IEEE), 894–901. doi:10.1109/ICRA.2017.7989110

Migimatsu, T. and Bohg, J. (2020). Object-centric task and motion planning in dynamic environments. *IEEE Robotics and Automation Letters* 5, 844–851

Muelling, K., Kober, J., and Peters, J. (2010). Learning table tennis with a mixture of motor primitives. In *2010 10th IEEE-RAS International Conference on Humanoid Robots* (IEEE), 411–416

Murali, A., Liu, W., Marino, K., Chernova, S., and Gupta, A. (2020). Same object, different grasps: Data and semantic knowledge for task-oriented grasping. In *Conference on Robot Learning*

Myers, A., Teo, C. L., Fermüller, C., and Aloimonos, Y. (2015). Affordance detection of tool parts from geometric features. In *2015 IEEE International Conference on Robotics and Automation (ICRA)* (IEEE), 1374–1381

Nabeshima, C., Kuniyoshi, Y., and Lungarella, M. (2007). Towards a model for tool-body assimilation and adaptive tool-use. In *2007 IEEE 6th International Conference on Development and Learning* (IEEE), 288–293

Nabeshima, C., Lungarella, M., and Kuniyoshi, Y. (2005). Timing-based model of body schema adaptation and its role in perception and tool use: A robot case study. In *Proceedings. The 4th International Conference on Development and Learning, 2005* (IEEE), 7–12

Nagata, F., Watanabe, K., and Izumi, K. (2001). Furniture polishing robot using a trajectory generator based on cutter location data. In *Proceedings 2001 ICRA. IEEE International Conference on Robotics and Automation (Cat. No. 01CH37164)* (IEEE), vol. 1, 319–324

Nair, L., Balloch, J., and Chernova, S. (2019a). Tool macgyvering: Tool construction using geometric reasoning. In *2019 International Conference on Robotics and Automation (ICRA)* (IEEE), 5837–5843

Nair, L., Srikanth, N. S., Erickson, Z. M., and Chernova, S. (2019b). Autonomous tool construction using part shape and attachment prediction. In *Robotics: Science and Systems*

Nakamura, T. and Nagai, T. (2010). Object concept modeling based on the relationship among appearance, usage and functions. In *2010 IEEE/RSJ International Conference on Intelligent Robots and Systems* (IEEE), 5410–5415

Nishide, S., Tani, J., Takahashi, T., Okuno, H. G., and Ogata, T. (2011). Tool–body assimilation of humanoid robot using a neurodynamical system. *IEEE transactions on autonomous mental development* 4, 139–149

Okada, K., Kojima, M., Sagawa, Y., Ichino, T., Sato, K., and Inaba, M. (2006). Vision based behavior verification system of humanoid robot for daily environment tasks. In *2006 6th IEEE-RAS International Conference on Humanoid Robots* (IEEE), 7–12

Paraschos, A., Daniel, C., Peters, J. R., and Neumann, G. (2013). Probabilistic movement primitives. *Advances in neural information processing systems* 26

Pastor, P., Hoffmann, H., Asfour, T., and Schaal, S. (2009). Learning and generalization of motor skills by learning from demonstration. In *2009 IEEE International Conference on Robotics and Automation* (IEEE), 763–768

Pastor, P., Kalakrishnan, M., Chitta, S., Theodorou, E., and Schaal, S. (2011). Skill learning and task outcome prediction for manipulation. In *2011 IEEE international conference on robotics and automation* (IEEE), 3828–3834

Peternel, L., Petrič, T., and Babič, J. (2015). Human-in-the-loop approach for teaching robot assembly tasks using impedance control interface. In *2015 IEEE international conference on robotics and automation (ICRA)* (IEEE), 1497–1502

Pfeiffer, K., Escande, A., and Kheddar, A. (2017). Nut fastening with a humanoid robot. In *2017 IEEE/RSJ International Conference on Intelligent Robots and Systems (IROS)* (IEEE), 6142–6148

Qin, M., Brawer, J., and Scassellati, B. (2021). Rapidly learning generalizable and robot-agnostic tool-use skills for a wide range of tasks. *Frontiers in Robotics and AI* 8

Qin, M., Brawer, J., and Scassellati, B. (2022). (under review) using task, affordance, and motion planning (taamp) to detect infeasible or limited solutions in affordance-constrained environments. *Under Review*

Qin, Z., Fang, K., Zhu, Y., Fei-Fei, L., and Savarese, S. (2020). Keto: Learning keypoint representations for tool manipulation. In *2020 IEEE International Conference on Robotics and Automation (ICRA)* (IEEE), 7278–7285

Raessa, M., Sánchez, D., Wan, W., Petit, D., and Harada, K. (2019). Teaching a robot to use electric tools with regrasp planning. *CAAI Transactions on Intelligence Technology* 4, 54–63

Ramirez-Amaro, K., Beetz, M., and Cheng, G. (2014a). Automatic segmentation and recognition of human activities from observation based on semantic reasoning. In *2014 IEEE/RSJ International Conference on Intelligent Robots and Systems* (IEEE), 5043–5048

Ramirez-Amaro, K., Beetz, M., and Cheng, G. (2015). Understanding the intention of human activities through semantic perception: observation, understanding and execution on a humanoid robot. *Advanced Robotics* 29, 345–362

Ramirez-Amaro, K., Inamura, T., Dean-León, E., Beetz, M., and Cheng, G. (2014b). Bootstrapping humanoid robot skills by extracting semantic representations of human-like activities from virtual reality. In *2014 IEEE-RAS International Conference on Humanoid Robots* (IEEE), 438–443

Robertsson, A., Olsson, T., Johansson, R., Blomdell, A., Nilsson, K., Haage, M., et al. (2006). Implementation of industrial robot force control case study: high power stub grinding and deburring. In *2006 IEEE/RSJ International Conference on Intelligent Robots and Systems* (IEEE), 2743–2748

Rozo, L., Jiménez, P., and Torras, C. (2013). Force-based robot learning of pouring skills using parametric hidden markov models. In *9th International Workshop on Robot Motion and Control* (IEEE), 227–232

Saito, N., Kim, K., Murata, S., Ogata, T., and Sugano, S. (2018). Tool-use model considering tool selection by a robot using deep learning. In *2018 IEEE-RAS 18th International Conference on Humanoid Robots (Humanoids)* (IEEE), 270–276

Sammut, C., Sheh, R., Haber, A., and Wicaksono, H. (2015). The robot engineer. In *ILP (Late Breaking Papers)*. 101–106

Schaal, S. (2006). Dynamic movement primitives-a framework for motor control in humans and humanoid robotics. In *Adaptive motion of animals and machines* (Springer). 261–280

Schoeler, M. and Wörgötter, F. (2015). Bootstrapping the semantics of tools: Affordance analysis of real world objects on a per-part basis. *IEEE Transactions on Cognitive and Developmental Systems* 8, 84–98

Shao, L., Migimatsu, T., Zhang, Q., Yang, K., and Bohg, J. (2021). Concept2robot: Learning manipulation concepts from instructions and human demonstrations. *The International Journal of Robotics Research* 40, 1419–1434

Sinapov, J. and Stoytchev, A. (2007). Learning and generalization of behavior-grounded tool affordances. In *2007 IEEE 6th International Conference on Development and Learning* (IEEE), 19–24

Sinapov, J. and Stoytchev, A. (2008). Detecting the functional similarities between tools using a hierarchical representation of outcomes. In *2008 7th IEEE International Conference on Development and Learning* (IEEE), 91–96. doi:10.1109/DEVLRN.2008.4640811

Song, D., Ek, C. H., Huebner, K., and Kragic, D. (2011a). Embodiment-specific representation of robot grasping using graphical models and latent-space discretization. In *2011 IEEE/RSJ International Conference on Intelligent Robots and Systems* (IEEE), 980–986

Song, D., Ek, C. H., Huebner, K., and Kragic, D. (2011b). Multivariate discretization for bayesian network structure learning in robot grasping. In *2011 IEEE International Conference on Robotics and Automation* (IEEE), 1944–1950

Song, D., Ek, C. H., Huebner, K., and Kragic, D. (2015). Task-based robot grasp planning using probabilistic inference. *IEEE transactions on robotics* 31, 546–561

Song, D., Huebner, K., Kyrki, V., and Kragic, D. (2010). Learning task constraints for robot grasping using graphical models. In *2010 IEEE/RSJ International Conference on Intelligent Robots and Systems* (IEEE), 1579–1585

Stoytchev, A. (2003). *Computational model for an extendable robot body schema*. Tech. rep., Georgia Institute of Technology

Stoytchev, A. (2005). Behavior-grounded representation of tool affordances. In *Proceedings of IEEE International Conference on Robotics and Automation (ICRA)* (IEEE), 3071–3076. doi:10.1109/ROBOT.2005.1570580

Stoytchev, A. (2008). Learning the affordances of tools using a behavior-grounded approach. In *Towards Affordance-Based Robot Control* (Springer). 140–158

Stückler, J. and Behnke, S. (2014a). Adaptive tool-use strategies for anthropomorphic service robots. In *2014 IEEE-RAS International Conference on Humanoid Robots* (IEEE), 755–760

Stückler, J. and Behnke, S. (2014b). Efficient deformable registration of multi-resolution surfel maps for object manipulation skill transfer. In *2014 IEEE International Conference on Robotics and Automation (ICRA)* (IEEE), 994–1001

Stückler, J. and Behnke, S. (2015). Perception of deformable objects and compliant manipulation for service robots. In *Soft Robotics* (Springer). 69–80

Stückler, J., Droeschel, D., Gräve, K., Holz, D., Schreiber, M., Topalidou-Kyniazopoulou, A., et al. (2013). Increasing flexibility of mobile manipulation and intuitive human-robot interaction in robocup@ home. In *Robot Soccer World Cup* (Springer), 135–146

Stückler, J., Schwarz, M., and Behnke, S. (2016). Mobile manipulation, tool use, and intuitive interaction for cognitive service robot cosero. *Frontiers in Robotics and AI* 3, 58

Su, Y.-H., Huang, K., and Hannaford, B. (2018). Real-time vision-based surgical tool segmentation with robot kinematics prior. In *2018 International Symposium on Medical Robotics (ISMR)* (IEEE), 1–6

Takahashi, K., Kim, K., Ogata, T., and Sugano, S. (2017). Tool-body assimilation model considering grasping motion through deep learning. *Robotics and Autonomous Systems* 91, 115–127

Takeuchi, Y., Ge, D., and Asakawa, N. (1993). Automated polishing process with a human-like dexterous robot. In *[1993] Proceedings IEEE International Conference on Robotics and Automation* (IEEE), 950–956

Tee, K. P., Cheong, S., Li, J., and Ganesh, G. (2022). A framework for tool cognition in robots without prior tool learning or observation. *Nature Machine Intelligence* , 1–11

Tee, K. P., Li, J., Chen, L. T. P., Wan, K. W., and Ganesh, G. (2018). Towards emergence of tool use in robots: Automatic tool recognition and use without prior tool learning. In *2018 IEEE International Conference on Robotics and Automation (ICRA)* (IEEE), 6439–6446

Tikhanoff, V., Pattacini, U., Natale, L., and Metta, G. (2013). Exploring affordances and tool use on the icub. In *2013 13th IEEE-RAS International Conference on Humanoid Robots (Humanoids)* (IEEE), 130–137. doi:10.1109/HUMANOIDS.2013.7029967

Toussaint, M. A., Allen, K. R., Smith, K. A., and Tenenbaum, J. B. (2018). Differentiable physics and stable modes for tool-use and manipulation planning. In *Robotics: Science and Systems Foundation*

Tsuji, T., Ohkuma, J., and Sakaino, S. (2015). Dynamic object manipulation considering contact condition of robot with tool. *IEEE Transactions on Industrial Electronics* 63, 1972–1980

Turpin, D., Wang, L., Tsogkas, S., Dickinson, S., and Garg, A. (2021). Gift: Generalizable interaction-aware functional tool affordances without labels. *arXiv preprint arXiv:2106.14973*

Vogel, J., Takemura, N., Höppner, H., van der Smagt, P., and Ganesh, G. (2017). Hitting the sweet spot: Automatic optimization of energy transfer during tool-held hits. In *2017 IEEE International Conference on Robotics and Automation (ICRA)* (IEEE), 1549–1556

Wicaksono, H. (2020). *A Relational Approach to Tool Creation by a Robot*. Ph.D. thesis, University of New South Wales, Sydney, Australia

Wicaksono, H. and Sammut, C. (2016). Relational tool use learning by a robot in a real and simulated world. In *Proceedings of ACRA*

Wölfel, K. and Henrich, D. (2018). Grounding verbs for tool-dependent, sensor-based robot tasks. In *2018 27th IEEE International Symposium on Robot and Human Interactive Communication (RO-MAN)* (IEEE), 378–383

Xie, A., Ebert, F., Levine, S., and Finn, C. (2019). Improvisation through physical understanding: Using novel objects as tools with visual foresight. In *Proceedings of Robotics: Science and Systems* (FreiburgimBreisgau, Germany). doi:10.15607/RSS.2019.XV.001

Xue, Y. and Jia, Y.-B. (2020). Gripping a kitchen knife on the cutting board. In *2020 IEEE/RSJ International Conference on Intelligent Robots and Systems (IROS)* (IEEE), 9226–9231

Yamazaki, K., Ueda, R., Nozawa, S., Mori, Y., Maki, T., Hatao, N., et al. (2010). System integration of a daily assistive robot and its application to tidying and cleaning rooms. In *2010 IEEE/RSJ International Conference on Intelligent Robots and Systems* (IEEE), 1365–1371

Zhu, Y., Zhao, Y., and Chun Zhu, S. (2015). Understanding tools: Task-oriented object modeling, learning and recognition. In *Proceedings of the IEEE Conference on Computer Vision and Pattern Recognition*. 2855–2864
